# Factors Associated with Appointment Cancellation on a Commercial Digital Mental Health Platform: A Retrospective Cohort Study in Saudi Arabia

**DOI:** 10.3390/healthcare14172672

**Published:** 2026-08-22

**Authors:** Abdulmajeed A. Alkhamees

**Affiliations:** Department of Psychiatry, College of Medicine, Qassim University, Buraidah 52571, Saudi Arabia; a.alkhamees@qu.edu.sa

**Keywords:** telemental health, digital mental health, appointment cancellation, no-show, health services, Saudi Arabia, cohort study, logistic regression

## Abstract

**Background:** Commercial digital mental health platforms have expanded across the Gulf since 2020, yet cancellation in this delivery model remains understudied. We aimed to identify factors independently associated with session cancellation on a Saudi Arabic-language commercial platform and to determine whether they describe user or provider behaviour. **Methods:** We conducted a retrospective cohort analysis of 36,544 booked sessions from 21,904 unique users (October 2023–April 2026); the outcome was binary cancellation (cancelled vs. completed). Multivariable logistic regression with cluster-robust standard errors estimated adjusted odds ratios (aORs) for user, provider, and booking characteristics; secondary analyses decomposed cancellation by recorded initiator and modelled age, lead time, and calendar time flexibly. Reporting followed STROBE guidelines. **Results:** Crude cancellation was 25.6%. Cancellation was strongly patterned by visit type (follow-up aOR 0.26, 95% CI 0.21–0.32), lead time (≥8 days 2.10, 95% CI 1.90–2.33), session type (free 1.19, 95% CI 1.10–1.27), and provider specialty; age reduced odds modestly (aOR 0.90 per decade, *p* < 0.001) and gender was null (aOR 0.95, *p* = 0.119). Cause-specific models showed that the lead-time gradient reverses by initiator: at ≥8 days versus same-day, user-initiated cancellation was less likely (aOR 0.62, 95% CI 0.49–0.78), whereas provider-initiated cancellation was far more likely (aOR 4.75, 95% CI 4.23–5.35), and provider-specialty differences were confined almost entirely to provider-initiated cancellation. The very low adjusted odds for emergency sessions (aOR 0.17, 95% CI 0.13–0.23) reflect how emergency bookings are administratively coded on the platform rather than genuine behavioural non-adherence and are regarded as artefactual. Discrimination was moderate (apparent AUC 0.700, development sample). **Conclusions:** Cancellation reflected operational features of the booking, but its largest correlates—lead time, free-session status, and provider specialty—operate predominantly through provider-side scheduling rather than patient non-adherence.

## 1. Introduction

Mental disorders affect a sizeable share of the Saudi adult population. The Saudi National Mental Health Survey, a nationally representative DSM-IV/CIDI face-to-face survey of 4004 citizens aged 15–65, estimated lifetime prevalence of any mental disorder at 34.2% and 12-month prevalence at 20.2%, with anxiety disorders being the most prevalent class [1,2]. National telephone-based screening conducted in 2022 found 12.7% of adults at risk for major depressive disorder and 12.4% at risk for generalised anxiety disorder, while only 1.5% and 0.5%, respectively, were diagnosed and treated [3]. Treatment-seeking remains low across the region, shaped by stigma, fragmented service supply, and limited mental health workforce capacity.

Digital mental health services have expanded across the Gulf since 2020. Saudi Arabia and the United Arab Emirates were among the first regional jurisdictions to issue telepsychiatry regulations [4,5]. During the COVID-19 pandemic, the Saudi Ministry of Health launched a national telemental health initiative recruiting volunteer mental health professionals to deliver synchronous remote care via secure platforms [6]. Subsequent national e-referral infrastructure (Saudi Medical Appointments and Referrals Centre) has begun coordinating mental health referrals nationwide [7]. Alongside these public-sector developments, commercial Arabic-language platforms now provide direct-to-consumer booking with licensed psychologists, psychiatrists, social and family counsellors, and coaches. The behavioural patterns of users on such platforms—and operationally, which sessions get kept and which get cancelled—remain poorly characterised in the published literature. This gap is notable given that patient engagement is itself increasingly treated as a multidimensional construct requiring explicit characterisation rather than assumption [8], and that digital mental health platforms face documented, modality-specific barriers to sustained user engagement [9].

Across all clinical specialties, missed appointments occur at an average rate of approximately 23% [10]. In mental health specifically, the proportion is higher: in 17,211 outpatient psychiatric contacts in the United Kingdom, non-attendance rates ranged from 5.0% in rehabilitation to 36.9% in alcohol services for new patients [11], and a contemporaneous review estimated that mental health appointment non-attendance is approximately twice the rate seen in other medical specialties, with up to half of patients who miss appointments dropping out of scheduled care [12]. Two systematic reviews—one synthesising 105 studies of no-shows across all specialties [10] and one focused on prediction models [13]—converge on lead time and prior no-show history as the strongest determinants. Most published prediction models, however, use clinic-based or hospital-based outpatient data; commercial digital platforms have different operational characteristics (immediate booking, free-tier acquisition, multi-provider-type matching), and the pattern of factors associated with cancellation may differ. Within Saudi Arabia and the wider Gulf specifically, prior work has examined missed-appointment factors in conventional hospital outpatient settings [14], machine-learning no-show prediction in general Saudi healthcare settings [15], and no-show patterns following telepsychiatry implementation in neighbouring Qatar [16]; none of these examined a privately operated, Arabic-language commercial digital mental health platform. The originality of the present study lies primarily in this platform-specific booking context rather than in the general concept of predicting or explaining missed appointments.

A recent meta-analysis of telehealth versus in-person care across multiple specialties reported reduced odds of non-attendance with virtual delivery (pooled OR 0.61) [17], but this body of evidence is dominated by post-2020 transitions in primarily public-sector or institution-based care. Cancellation behaviour on a privately operated, fully online, Arabic-first platform is not directly addressed by these data. The present study uses 36,544 booking records from a single Saudi commercial digital mental health platform to estimate adjusted associations between user, provider, and operational factors and the odds that a scheduled session is cancelled rather than attended. The primary objective was explanatory—identifying factors independently associated with recorded cancellation—rather than the development or validation of a prediction model. A secondary, descriptive objective was to summarise how well these factors jointly separated cancelled from completed bookings.

## 2. Materials and Methods

### 2.1. Setting and Platform

Talbinah is a Saudi Arabia-based commercial Arabic-language digital mental health platform offering session booking with licensed psychologists, psychiatrists, social and family counsellors, family-medicine and general-practice physicians, and coaches. Users register on the platform, browse providers, and book sessions in synchronous video format. The platform offers several pricing tiers including standard paid sessions, package-based subscriptions, free-tier sessions (used predominantly for acquisition and first-touch encounters), free-AI assistant interactions, and emergency sessions. All booking and cancellation events are logged to the platform database with timestamps, provider specialty, and session metadata.

Free-AI sessions warrant separate description because their operational profile differs from other session types. These are brief, AI-assisted triage interactions: an initial request is assessed via an AI-assisted intake process, and the user is then connected with a live human provider (95.3% psychiatrists, 4.7% coaches, in this sample) for a short consultation (median duration 5 min; maximum 30 min). Free-AI bookings are almost always same-day (91.7% with zero-day lead time, versus a mean of several days for other session types) and are booked, recorded, and cancelled through the same platform mechanism as other sessions, with no separate booking pathway or completion criterion. Cancellation of Free-AI sessions is predominantly user-initiated (62.4% of cancelled Free-AI bookings, versus 38.3% user-initiated across all session types), and the row-wise cancellation rate is 14.5% (348/2403). Because Free-AI interactions are staffed by the same licensed providers and follow the same booking and cancellation pipeline as professional sessions, they were retained in the primary model as a distinct session-type category rather than excluded or modelled separately; their markedly shorter duration and near-zero lead time are noted as a limitation on direct comparability with standard sessions (Section 4.4). Free-AI sessions were also introduced at a specific point in the platform’s history, and the apparently protective adjusted association for this category is not robust to adjustment for calendar time (Section 3.6); we therefore do not report it as a finding.

Emergency sessions likewise require clarification. This category is an expedited-access booking option rather than a distinct, clinically governed psychiatric emergency service: bookings are fulfilled by providers across the full specialty mix present on the platform (54.5% psychologists, 26.3% psychiatrists, 10.5% family GPs, 6.3% social/family counsellors, 2.5% coaches, in this sample), are booked almost always for same-day delivery (94.5% zero-day lead time), and are of typical duration for a standard consultation (median 30 min, interquartile range 30–45 min). The platform’s specific triage criteria for routing a request into this category, and whether a distinct crisis-escalation pathway exists outside ordinary booking, could not be determined from the data extract and are noted as a limitation (Section 4.4) rather than asserted. Recorded cancellation of emergency sessions is rare under the primary status_label definition (6.3%, 72/1140) and could not be reliably re-estimated under the was_cancelled flag (only 1 of these 72 bookings carried that flag), producing quasi-complete separation; as reported in Section 3.4 and Section 3.7, essentially all of these recorded cancellations are administrative status changes with no cancellation record and no recorded initiator, and the adjusted estimate for this category is treated as artefactual rather than behavioural throughout.

### 2.2. Study Design and Data Source

This is a retrospective cohort analysis at the level of the booked session. The data extract spans bookings created between October 2023 and April 2026. Two linked anonymised datasets were used: a session-booking table (47,240 booking records) and a user-level demographics table (37,004 users). Linkage was performed on a SHA-256-hashed user identifier. The data extract was provided by the platform under a data-governance agreement; no re-identifiable information was accessed by the analyst.

### 2.3. Inclusion and Exclusion Criteria

**Figure 1:**
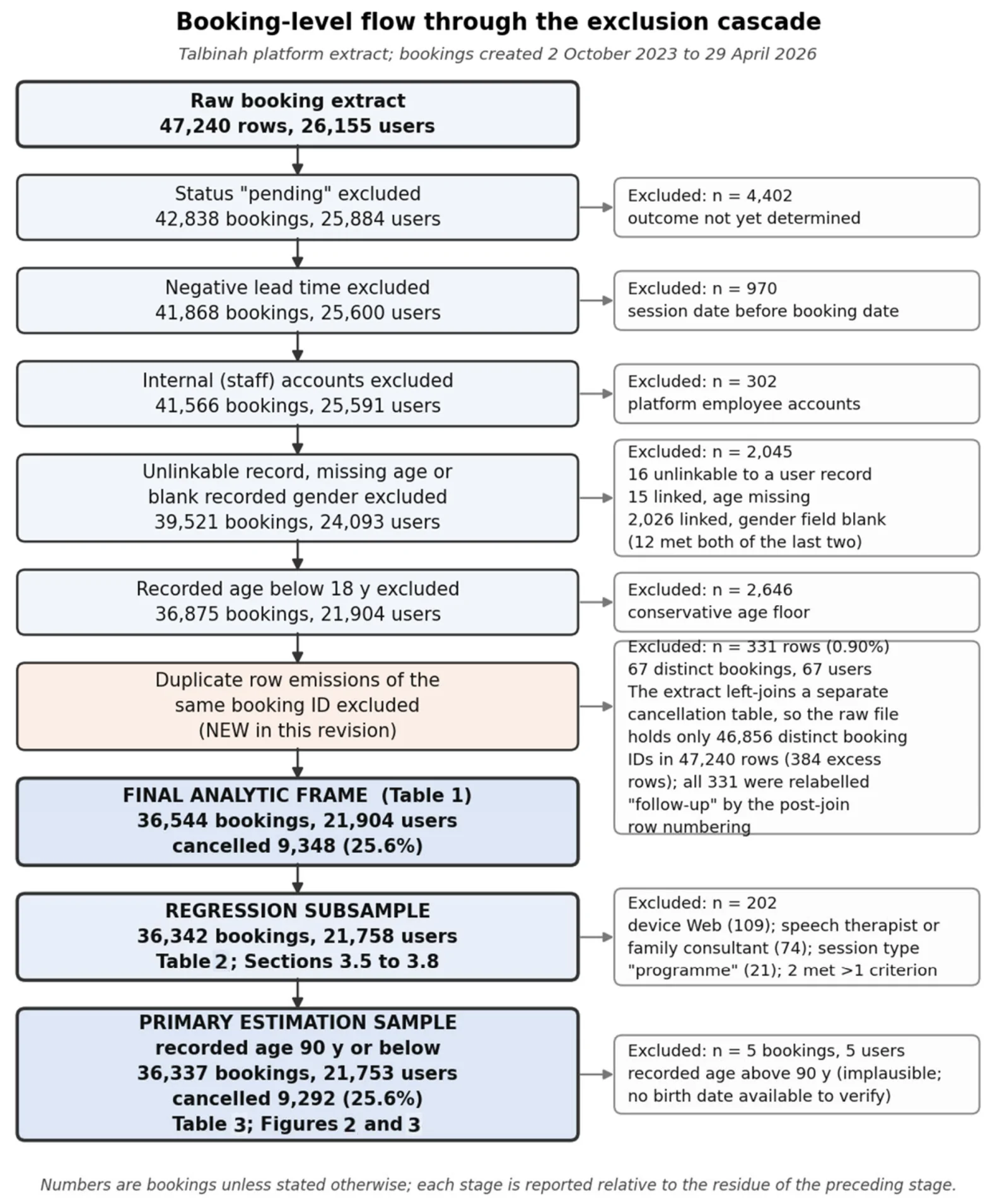
Booking-level flow through the exclusion cascade. Each stage is reported relative to the residue of the preceding stage. The sixth step, removal of duplicate row emissions of the same booking identifier, was added in the present revision (Section 2.3). The final analytic frame (36,544 bookings, 21,904 users) is described in Table 1; the regression subsample (36,342 bookings, 21,758 users) is the estimation frame for Table 2 and for Section 3.5, Section 3.6, Section 3.7 and Section 3.8; the primary estimation sample after the age restriction (36,337 bookings, 21,753 users) is the estimation frame for Table 3 and Figure 2 and Figure 3.

Exclusions were applied sequentially to the raw extract of 47,240 booking records (26,155 unique users), and each stage is reported relative to the residue of the preceding stage. Bookings with status “pending” were excluded as the cancellation outcome was not yet determined at extract time (n = 4402; 42,838 bookings, 25,884 users remaining). Bookings with negative lead time (session_date earlier than booking_created_at) were excluded as data anomalies (n = 970; 41,868 bookings, 25,600 users remaining). Internal (platform employee) accounts were excluded (n = 302; 41,566 bookings, 25,591 users remaining). Bookings that could not be linked to a valid user record, or for which age was missing or recorded gender was outside “Female”/“Male” (the only two values present in the source field), were excluded (n = 2045; 39,521 bookings, 24,093 users remaining); of these 2045 bookings, 16 could not be linked to any user-level record, 15 were linked but had a missing age value, and 2026 were linked but had a blank recorded-gender field (12 bookings met both of the latter two criteria). As a conservative measure discussed in the Ethics Statement, bookings linked to users with a recorded age below 18 years were excluded (n = 2646; 36,875 bookings, 21,904 users remaining). Finally, a sixth step added in the present revision removed duplicate row emissions of the same anon_booking_id (n = 331; 0.90% of the preceding residue), giving a final analytic frame of 36,544 bookings from 21,904 unique users (median bookings per user = 1; interquartile range 1–1; mean 1.67; maximum 207). Table 1 reports characteristics of the resulting analytic sample by cancellation status, not the exclusion flow itself; a STROBE-style flow diagram reproducing this six-step cascade, with the number of bookings and unique users remaining at every stage and the reason for every exclusion, is provided as Figure 1.

The sixth exclusion step requires explicit disclosure, because it corrects an artefact that affected two of the previously reported estimates. The anonymised booking identifier is not unique in the source extract: the extraction query left-joins a separate cancellation table to the booking table, so a booking associated with more than one cancellation record is emitted once per cancellation record, and the row-numbering window functions that generate the booking-sequence and visit-type variables are evaluated after that join. Every duplicate emission after the first therefore received an incremented sequence number and was relabelled as a follow-up visit. In the previously reported analytic frame of 36,875 bookings, 331 rows (0.90%), covering 67 distinct bookings from 67 users, were such duplicate emissions; all 331 carried visit type “follow-up”, 298 were recorded as cancelled, 329 carried a provider-initiated cancellation record, and two single bookings accounted for 54 and 36 rows respectively. The affected cells were highly concentrated: 298 of the 1453 “follow-up visit and cancelled” bookings (20.5%) and 245 of the 1886 “device Unknown and cancelled” bookings (13.0%) were duplicate emissions, against 3.1% of all cancelled bookings. This is an artefact of the data extract rather than of the platform’s booking records. It was identified during the present revision by verifying the uniqueness of the booking identifier; the de-duplicated sample is used as the primary analysis throughout, all counts, tables and figures have been regenerated from it, and the consequences for the previously reported device-type and follow-up-visit estimates are reported in Section 3.2 and revisited in Section 4.4.

Missing data were handled by exclusion, not imputation, and are reported explicitly rather than left implicit. Age and recorded gender are complete (0% missing) in the final analytic frame by construction, because records with missing age or a gender value outside {Female, Male} were excluded at the fourth exclusion step above (n = 2045); no imputation was performed for these variables. Recorded age was additionally checked for plausibility: values ranged from 18 to 132 years, with 76 bookings from 68 users (0.21%) at 70 years or above and 10 bookings from 10 users (0.03%) at 90 years or above, of which 6 bookings from 6 users (0.02%) were strictly above 90 years; the single highest value, 132 years, was one booking from one user. The extract contains no date-of-birth field, so implausible values cannot be recomputed against a birth date and verification was limited to range checking; this is stated rather than implying that the source records were audited. For the primary regression, recorded ages strictly above 90 years were excluded as implausible, which removes 5 of those 6 bookings because the single 132-year record had already been excluded from the regression subsample on an earlier criterion, having been placed from the web client (Section 2.5): 90 years exceeds the 99.9th percentile of the recorded age distribution in the analytic frame (77.5 years) by more than 12 years and is well above Saudi period life expectancy, so the bound cannot plausibly exclude real users. The six bookings recorded above 90 years carry ages of 95 (two bookings), 97, 98, 99 and 132 years. Device type is missing for 13.7% of bookings in the final sample (4999/36,544); rather than imputing or dropping these records, device type was retained with an explicit “Unknown” category (Table 1 and Table 3). “Unknown” was retained because it represents a distinct data-capture state in the platform, not because of any inference about the missing-data mechanism; we make no claim that these data are missing at random, missing not at random, or otherwise, since the available fields cannot identify the mechanism. Cause-specific analyses (Section 3.4) indicate that Unknown device type is associated almost exclusively with provider-initiated and administratively recorded cancellations rather than with user-initiated cancellation, consistent with it marking a platform-side record state. The cancelled_by field is structurally populated only for cancelled bookings and is therefore missing platform-wide for the 74.4% of bookings that were completed; among cancelled bookings specifically, 15.2% lack a recorded initiator (Section 2.4). No other covariate used in the descriptive or regression analyses had missing values in the final analytic sample.

### 2.4. Outcome Definition

Cancellation, late cancellation, no-show, non-attendance, rescheduling, and treatment dropout are related but conceptually distinct events, with different behavioural and operational meanings: a booking cancelled early enough for the slot to be reassigned is not equivalent to a no-show, where a user fails to attend without notice; a provider-initiated cancellation reflects clinic-side scheduling rather than user disengagement; and dropout describes discontinuation across multiple sessions rather than the fate of a single booking. This study examines only the first of these—recorded cancellation of an individual booking, as captured by the platform’s own status field—rather than no-show, rescheduling, or dropout, and rather than a composite measure spanning several of these constructs.

The primary outcome was binary cancellation, defined as platform status_label = “cancelled” (numeric status code 0). Bookings with status_label = “completed” (status code 1) constituted the comparator. We additionally examined an internal flag (was_cancelled = 1, derived from a separate cancel_reservations table); on the final analytic sample, 7962 bookings carried this flag versus 9348 with status_label = “cancelled”. The two definitions disagree for 1422 bookings: 1404 were marked cancelled by the status field without carrying the flag, and 18 carried the flag but were ultimately marked completed. The net difference in the two column totals is 1386, which is not the same quantity as the number of discordant records; the figure of 1353 reported in the previous version was this net difference on the pre-de-duplication sample and conflated the two and is withdrawn. All 1404 status-cancelled bookings without the flag also have a missing cancelled_by value, and this is structural rather than coincidental: both the flag and the initiator field are derived from the same cancel_reservations join, so a booking with no cancellation row necessarily has both was_cancelled = 0 and cancelled_by missing. The “cancellations with no recorded initiator” and the “cancelled-but-unflagged” records are therefore very largely the same bookings described twice (1404 of 1417), rather than two independent measurement puzzles. Their composition by session type is free 777, package 217, standard 193, free-AI 130, emergency 71, supported 9, and programme 7. The 18 records in the opposite direction all carry a provider-initiated cancellation record on a booking the platform ultimately marked completed, most plausibly cancelled-then-reinstated or rescheduled-in-place bookings. Using the cancelled_by field, populated for 84.8% of status_label-cancelled bookings, the initiator was the treating provider in 4343 cases (46.5%), the user in 3576 (38.3%), and an administrator in 12 (0.1%); no initiator was recorded for 1417 (15.2%). For the primary analysis we used status_label as the more inclusive definition. A cause-specific decomposition by recorded initiator, which is the principal new analysis in this revision, is reported in Section 3.4, and sensitivity analyses using the restrictive flag and a strictly concordant case definition are reported in Section 3.7.

### 2.5. Study Variables

Nine covariate blocks were specified a priori for the primary model, based on the published no-show literature [10,13] and platform operational characteristics:

- Gender (female vs. male, the only categories present).
- Age in years, modelled per 10-year increment.
- Device type at registration (Android [reference], iOS, unknown).
- Provider specialty (psychologist [reference], psychiatrist, social/family counsellor, family GP, coach).
- Session type (normal/standard [reference], free, package, free-AI, emergency, supported).
- Visit type (first [reference], follow-up with same provider, new provider).
- Lead time category in days between booking creation and scheduled session: same-day [reference], 1 day, 2–3 days, 4–7 days, ≥8 days.
- Log-transformed booking sequence number.
- Cluster: anonymised user identifier (for cluster-robust standard errors).

Bookings with device_type = “Web” (n = 109, 0.3% of the analytic frame; 89 unique users), with a speech-therapist or family-consultant provider (n = 74, 0.2%), or with session_type = “programme” (n = 21, <0.1%) were dropped from the regression subsample for stability rather than retained as low-information indicator categories (202 bookings excluded in total, of which 2 met more than one criterion). This defines the “regression subsample”: n = 36,342 bookings from 21,758 users. Recorded ages above 90 years were then excluded as implausible (n = 5 bookings from 5 users; see Section 2.3), which defines the “primary estimation sample” used for Table 3 and the spline analyses: n = 36,337 bookings from 21,753 users. The only difference between the two frames is that age-cleaning step, and it is the sole reason the booking count falls from 36,342 to 36,337 and the user count from 21,758 to 21,753; the two labels are used consistently throughout, and every table and figure caption identifies which of the two frames were used. The cause-specific models (Table 2) and the prior-history, calendar-time, alternative-outcome and sparsity analyses (Section 3.5, Section 3.6, Section 3.7 and Section 3.8) were estimated on the regression subsample of 36,342 bookings, because they do not turn on the age variable; the age restriction changes no coefficient in Table 3 by more than 0.002 on the odds-ratio scale, so the implausible values were identified, quantified and excluded but are not claimed to have biased the earlier estimates.

Three further covariate sets were derived for the secondary analyses. First, cancellation initiator was taken from the cancelled_by field and used to define a four-level outcome (completed; user-initiated cancellation; provider-initiated cancellation; administrative or no recorded initiator) for the cause-specific models in Section 3.4. Second, prior attendance and cancellation history were derived within each user from the full raw booking table, including records later excluded and bookings still pending, because a user’s booking history is a property of their record rather than of the analytic subset. Only information that had already resolved before the index booking was created was allowed to contribute: the outcome of the immediately preceding resolved booking (no resolved prior booking, completed, or cancelled), the number of prior cancellations (top-coded at five), and the proportion of prior bookings cancelled. Resolution date was taken as the cancellation date for cancelled bookings, falling back to the session date where the cancellation date was missing, and as the session date for completed bookings; pending bookings never contributed. A deliberately leakage-prone variant, in which every earlier booking contributed regardless of when its outcome became known, was fitted only to demonstrate the size of the leakage. Within-user chronological ordering is unambiguous: the platform’s booking-sequence field is a row number over the full creation timestamp, whereas the exported creation date is truncated to the day, and the two orderings agree for 100.0% of the 47,240 raw records. Third, calendar variables were derived from the exported dates: calendar year, quarter and month of booking creation, weekday of the scheduled session, and scheduled time of day. The clock time at which a booking was placed is the only temporal field genuinely unavailable, because the booking-creation timestamp is date-truncated in the extract.

### 2.6. Statistical Analysis

A multivariable logistic regression model was estimated using maximum likelihood with cluster-robust standard errors clustered at the user level, following the framework described by Cameron and Miller [18]. All covariates were entered simultaneously; no stepwise selection was performed. Adjusted odds ratios (aORs) and 95% confidence intervals are reported. As a descriptive, exploratory summary of how well the covariates jointly separated cancelled from completed bookings, the apparent area under the receiver operating characteristic curve (AUC) was calculated in the analysis sample. Because the objective was the estimation of adjusted associations rather than the development of a prediction model, no internal, temporal, or external validation was undertaken, and the apparent AUC is not an estimate of performance in new data. No predictive model is proposed, developed, or evaluated in this paper.

Five sets of secondary analyses were added in the present revision, each addressing a specific methodological concern. First, cancellation was decomposed by recorded initiator using three cause-specific binary logistic models, each comparing one cancellation type against completed bookings only, with the identical covariate set and user-level cluster-robust standard errors. A four-level multinomial model was fitted as confirmation; with the full covariate set it is not identified, because three session-type categories contain no provider-initiated cancellations at all, so it is reported after pooling the four sparse session types. For covariate categories that are separated in the provider-initiated model, Firth penalised logistic regression with profile penalised-likelihood confidence intervals was fitted alongside the unpenalised cluster-robust estimates; Firth intervals are not cluster-robust, because penalised likelihood admits no closed-form sandwich estimator, and are reported alongside rather than instead of them. Second, the linearity of the age and lead-time associations on the logit scale was tested by refitting the model with Harrell restricted cubic splines, in which the first basis column is the linear term so that linearity is a joint restriction on the remaining columns; the primary test is a cluster-robust joint Wald test, with the naive likelihood-ratio test reported alongside. Age knots were placed at the recommended quantiles; lead-time knots were pre-specified on the day scale, because 53% of bookings are same-day and quantile-based knots would coincide. Third, prior attendance and cancellation history were added to the primary model to quantify attenuation of the visit-type and session-type coefficients. Fourth, calendar time was added as a smooth restricted-cubic-spline trend in months since the start of the extract window, together with the weekday of the scheduled session. Fifth, the outcome definition was varied, and covariate overlap and sparsity were examined by cross-tabulation, variance-inflation factors, generalised variance-inflation factors by covariate block, and refitting after excluding the most structurally dependent session types. Wilson score intervals are reported for within-category crude cancellation rates. Analyses were conducted in Python 3.11 using pandas 3.0.2, numpy 2.4.4, statsmodels 0.14.6, and patsy 1.0.2.

### 2.7. Reporting

Reporting follows the STROBE guidelines for cohort studies [19]. A completed STROBE checklist is provided as Supplementary Material.

## 3. Results

### 3.1. Sample Characteristics

The analytic frame comprised 36,544 booked sessions placed by 21,904 unique users over a 31-month period (bookings created from 2 October 2023 to 29 April 2026). Crude cancellation was 25.6% (9348/36,544). Median user age was 28 years (IQR 22–35; mean 29.2, SD 8.9); the recorded distribution extended to an implausible maximum of 132 years, contributed by a single booking from a single user, and 10 bookings (0.03%) recorded an age of 90 years or above, 6 of them strictly above 90 years. These values were quantified, and the ages strictly above 90 years were excluded from the primary regression rather than merely flagged (n = 5 bookings within the regression subsample; Section 2.3 and Section 2.5). Recorded female gender accounted for 69.1% of bookings. Most bookings were made via an iOS (73.7%) or Android (12.3%) device; the device was unknown for 13.7%, and 0.3% used the web client. Provider distribution was skewed toward psychologists (64.0%), with psychiatrists (16.4%), social/family counsellors (14.6%), family GPs (3.3%), coaches (1.5%), and the two smallest categories, speech therapists (0.2%) and family consultants (<0.1%), making up the remainder. Same-day booking was the modal pattern (53.4% of bookings), with a broadly declining frequency at longer lead times. First visits were 58.8% of bookings, follow-ups 28.6%, and new-provider visits 12.6%. Free sessions accounted for 45.8% of bookings, standard paid sessions 37.5%, free-AI sessions 6.6%, package sessions 6.6%, emergency sessions 3.1%, supported sessions 0.5%, and programme sessions < 0.1%. Most users contributed a single booking (17,671 of 21,904; median 1, IQR 1–1), but 4233 of the 21,904 users (19.3%) contributed more than one, and those users account for 18,873 of the 36,544 bookings (51.6%), the maximum for any one user being 207. Detailed sample characteristics by cancellation status, with within-category cancellation rates and Wilson 95% confidence intervals, are presented in Table 1.

### 3.2. Factors Associated with Cancellation After Adjustment

The fitted multivariable logistic regression had a McFadden (likelihood-ratio-based) pseudo-R^2^ of 0.085 and converged in fewer than 25 iterations. Adjusted odds ratios are presented in Table 3 and visually in Figure 4. After adjustment for all other covariates, recorded female gender carried no statistically reliable association with cancellation (aOR 0.95, 95% CI 0.88–1.01, *p* = 0.119), in contrast to the modest crude difference observed in Table 1. Each additional decade of age was associated with 10% lower odds of cancellation (aOR 0.90, 95% CI 0.86–0.93, *p* < 0.001), consistent with the direction reported in the broader no-show literature [10]. This per-decade figure is a descriptive average across the observed age range: as Section 3.3 shows, the association is concentrated in early adulthood, so a single per-decade odds ratio understates risk in the youngest users and overstates the protective effect of older age.

Provider specialty showed substantial adjusted associations with cancellation. Compared with psychologists (the modal category), psychiatrists (aOR 0.56, 95% CI 0.50–0.62), social/family counsellors (aOR 0.61, 95% CI 0.56–0.67) and coaches (aOR 0.45, 95% CI 0.35–0.57) had lower adjusted cancellation odds, while the estimate for family GPs was no longer statistically reliable after de-duplication (aOR 0.88, 95% CI 0.77–1.01, *p* = 0.066). These specialty contrasts should not be read as differences in user behaviour: as Section 3.4 shows, they are confined almost entirely to provider-initiated cancellation, and because the extract contains no de-identified provider key they may substantially reflect clustering of cancellation practice within individual providers rather than any property of the specialty. Session type also strongly differentiated cancellation. Emergency sessions had the lowest adjusted odds (aOR 0.17, 95% CI 0.13–0.23), package sessions next (aOR 0.47, 95% CI 0.36–0.63), then free-AI sessions (aOR 0.58, 95% CI 0.49–0.69). The emergency estimate rests on a recording artefact rather than on behaviour and is not interpreted substantively (Section 3.4 and Section 3.7), and the free-AI estimate is explained by calendar time (Section 3.6). Free sessions had higher adjusted odds than standard paid sessions (aOR 1.19, 95% CI 1.10–1.27, *p* < 0.001). Follow-up visits with the same provider had 74% lower odds than first visits (aOR 0.26, 95% CI 0.21–0.32, *p* < 0.001), while booking a new provider after a prior one was not associated with cancellation (aOR 0.99, 95% CI 0.83–1.18).

Cancellation odds rose with the interval between booking and scheduled session, but the categorical coding in Table 3 summarises a continuous gradient rather than a threshold. A one-day lead time carried slightly lower odds than same-day booking (aOR 0.87, 95% CI 0.81–0.94) and 2–3 days was indistinguishable from same-day (aOR 0.98, 95% CI 0.90–1.07); lead times of 4–7 days raised cancellation odds (aOR 1.26, 95% CI 1.15–1.37) and lead times of eight or more days approximately doubled them (aOR 2.10, 95% CI 1.90–2.33, *p* < 0.001). Section 3.3 shows, using restricted cubic splines, that this is a smooth and continuous rise rather than a discrete threshold at eight days, and Section 3.4 shows that the pooled gradient is a weighted average of associations running in opposite directions for user-initiated and provider-initiated cancellation. The log-transformed booking sequence number was not associated with cancellation after adjustment for visit type and other covariates (aOR 1.00, 95% CI 0.88–1.13). Device type was not associated with cancellation after de-duplication: Unknown versus Android gave aOR 1.05 (95% CI 0.95–1.16, *p* = 0.312) and iOS versus Android aOR 1.01 (95% CI 0.93–1.10, *p* = 0.841). The 26% elevation for Unknown device reported in the previous version (aOR 1.26, 95% CI 1.09–1.46) was an artefact of the duplicated cancellation rows described in Section 2.3, 13.0% of which fell in the Unknown-device cancelled cell; that association is withdrawn. Removing the duplicates strengthened the follow-up-visit association in the opposite direction, from aOR 0.35 (0.29–0.42) to 0.26 (0.21–0.32), and improved model fit (pseudo-R^2^ 0.078 to 0.085; apparent AUC 0.690 to 0.700). Every other coefficient moved within its previous confidence interval.

### 3.3. Flexible Modelling of Age and Lead Time

**Figure 2:**
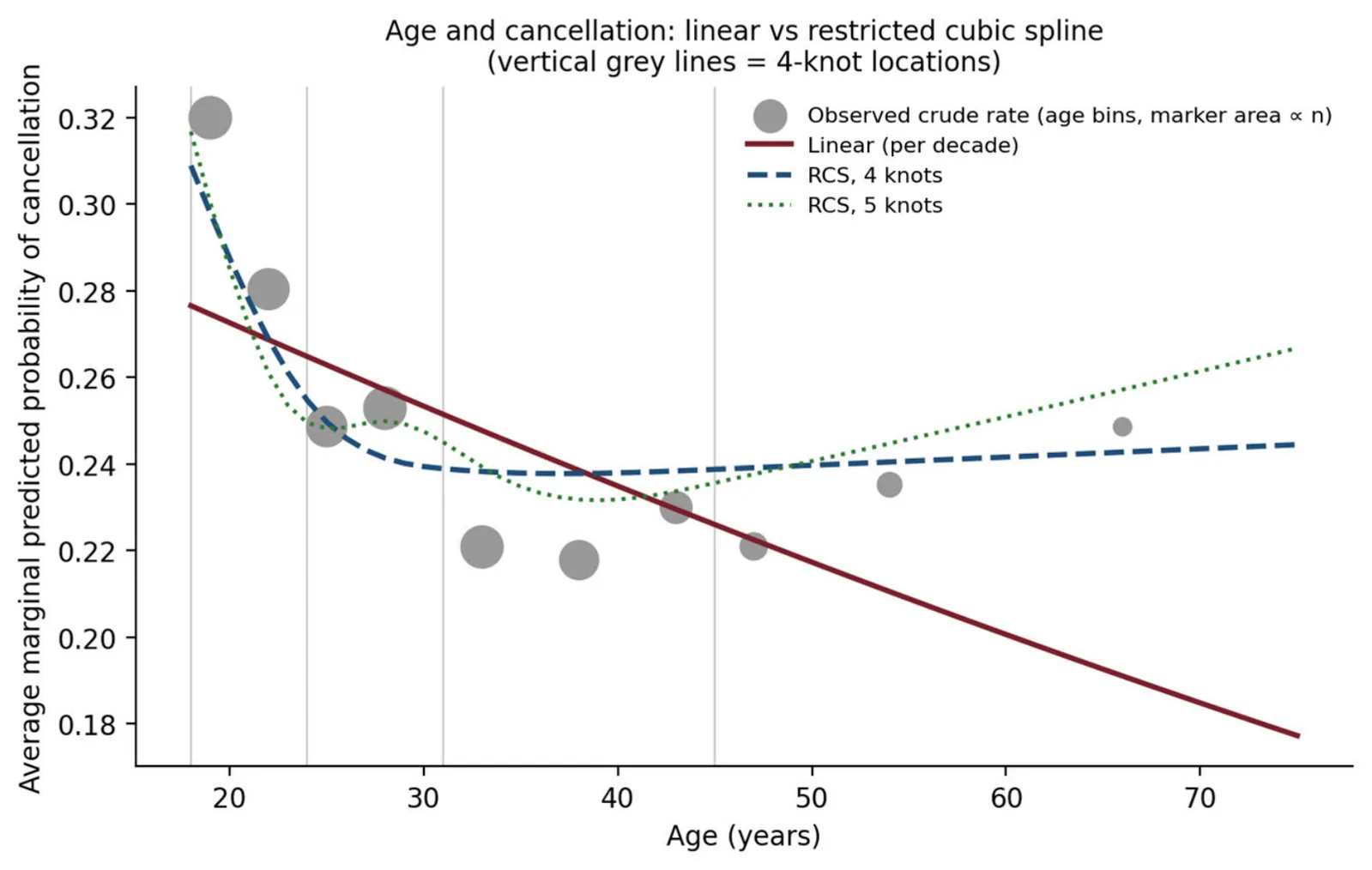
Average marginal predicted probability of cancellation as a function of age, comparing the linear per-decade specification with three- to five-knot restricted cubic splines (primary estimation sample: n = 36,337 bookings, 21,753 users).

Linearity of the age association on the logit scale was tested by replacing the per-decade term with a four-knot restricted cubic spline. Linearity was rejected (cluster-robust joint Wald χ^2^ = 29.02, df = 2, *p* = 5.0 × 10^−7^; naive likelihood-ratio χ^2^ = 48.42, df = 2, *p* = 3.1 × 10^−11^; ΔAIC = −44.4 in favour of the spline). The fitted shape is a steep decline followed by a plateau rather than a constant per-decade decrement. Average marginal predicted cancellation probability fell from 0.309 at age 18 to 0.250 at 25 years and was then essentially flat across the remainder of the range (0.239 at 30, 0.238 at 35 and at 40, 0.240 at 50, 0.242 at 60 and 0.244 at 70 years), whereas the linear per-decade specification imposes a continuing decline to 0.185 by age 70. The association between age and cancellation is therefore concentrated in early adulthood: risk declines steeply between 18 and approximately 25 years and is flat thereafter. The per-decade odds ratio of 0.90 in Table 3 is retained as a descriptive average and should be read as such rather than as a constant gradient (Figure 2).

**Figure 3:**
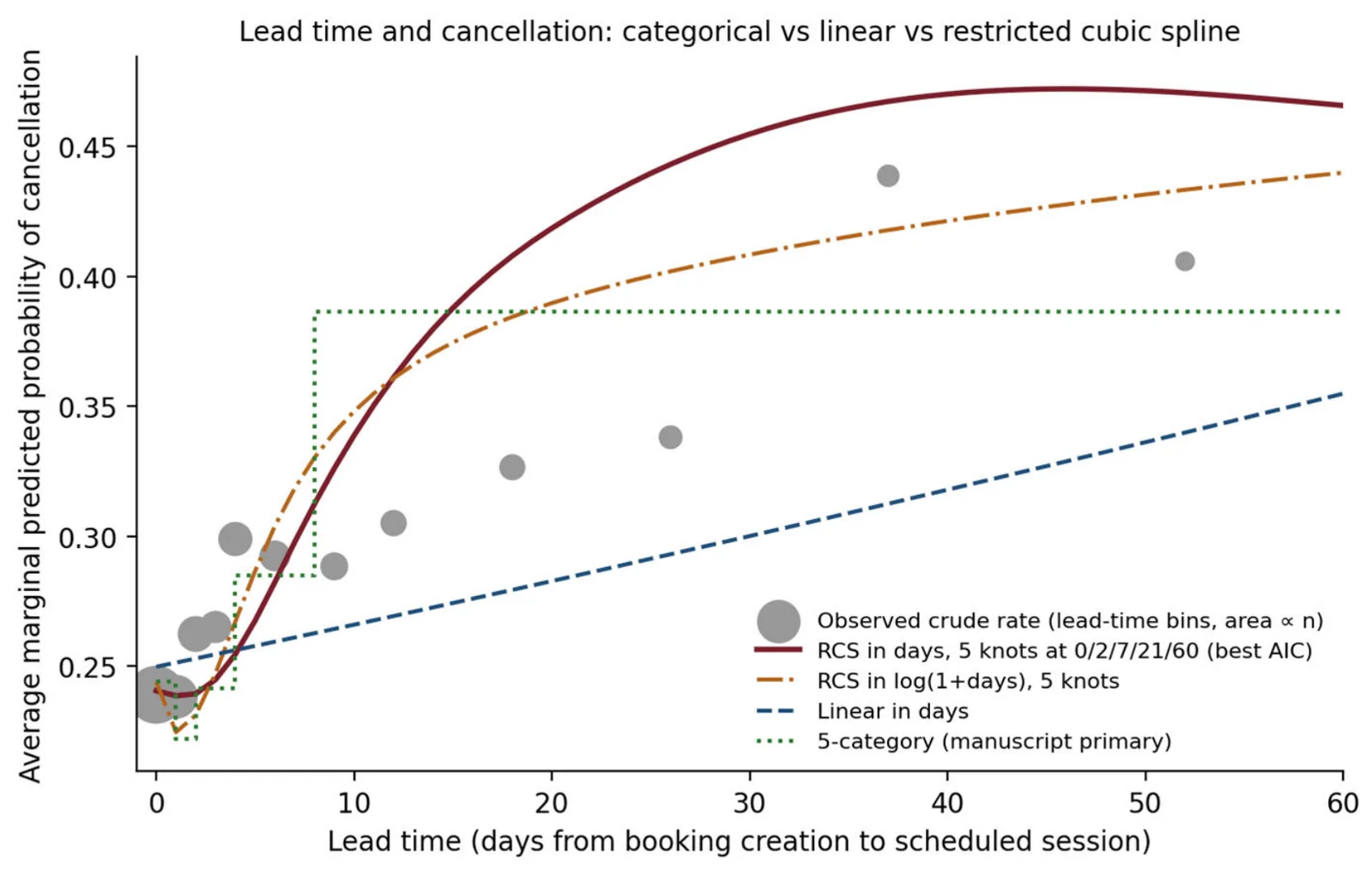
Average marginal predicted probability of cancellation as a function of lead time in days, comparing restricted cubic splines on the day and log (1 + days) scales with the linear and five-category specifications (primary estimation sample: n = 36,337 bookings, 21,753 users). The categorical coding is shown as a step function to make the artefactual nature of the apparent eight-day threshold visible.

**Figure 4:**
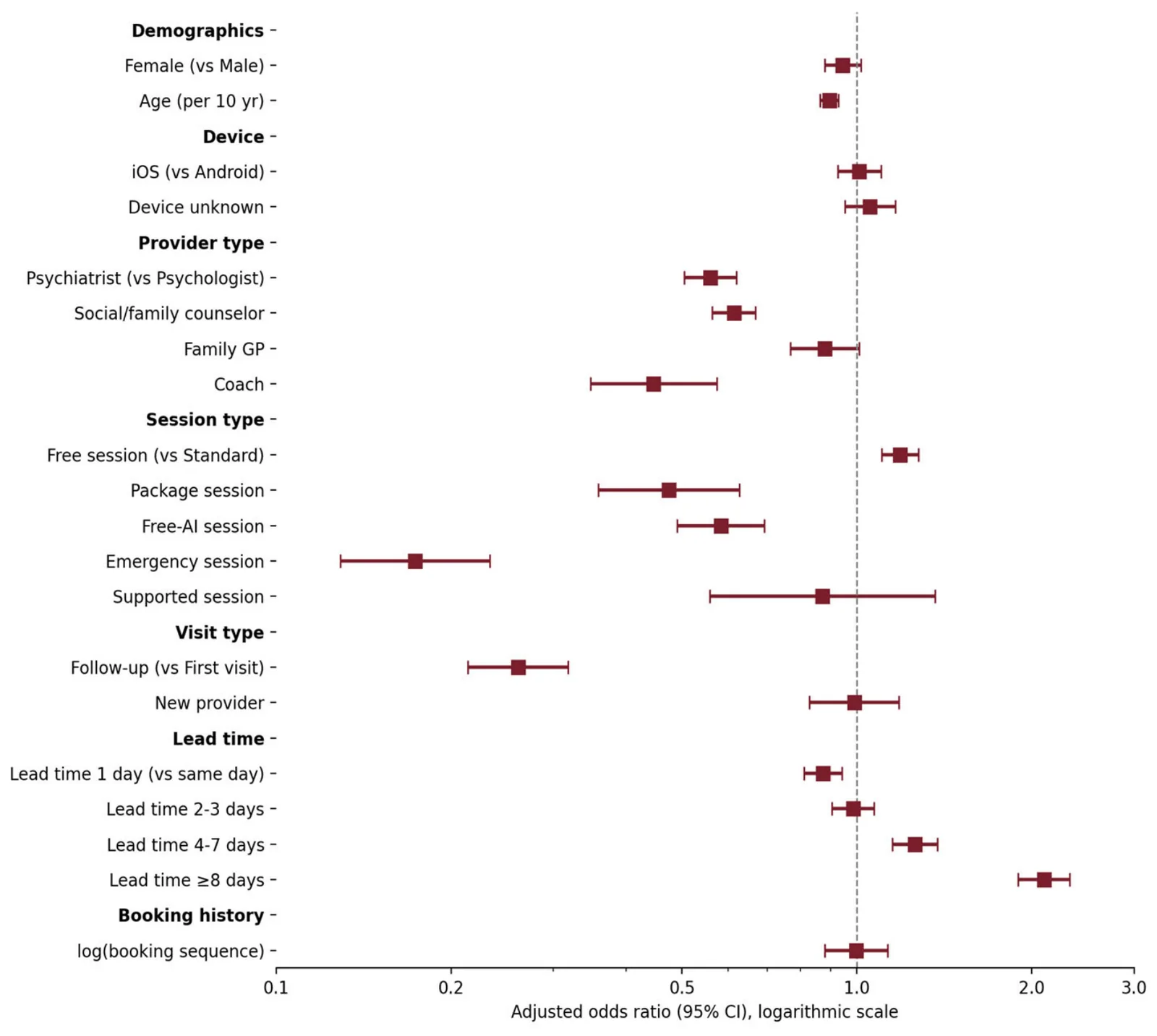
Adjusted odds ratios with 95% confidence intervals for booking cancellation, grouped by covariate block. Horizontal axis is logarithmic; the reference category for each block is named in parentheses next to the first row of that block (e.g., “vs. Male”, “vs. Android”, “vs. Psychologist”); marker colour is uniform and does not encode statistical significance, which can be read from whether each interval crosses the dashed reference line at 1.0.

Lead time was modelled continuously with a five-knot restricted cubic spline on the day scale, with knots pre-specified at 0, 2, 7, 21 and 60 days. Linearity in days was decisively rejected (cluster-robust joint Wald χ^2^ = 168.17, df = 3, *p* = 3.2 × 10^−36^; naive likelihood-ratio χ^2^ = 237.54, df = 3, *p* = 3.2 × 10^−51^), and the spline also fitted better than the five-category coding used in Table 3 (ΔAIC = −30.8). Crucially, the fitted curve shows no discontinuity at eight days. Average marginal predicted cancellation probability was flat over the first three days after booking (0.241 same-day, 0.239 at 1 day, 0.239 at 2 days, 0.245 at 3 days) and then rose smoothly and monotonically: 0.255 at 4 days, 0.298 at 7 days, 0.312 at 8 days, 0.339 at 10 days, 0.380 at 14 days, 0.423 at 21 days and 0.455 at 30 days, flattening near 0.47 beyond about six weeks. A parallel spline on the log (1 + days) scale produced the same shape. We therefore withdraw the description of this association as “threshold-like”: there is no threshold at eight days, and the apparent step in the categorical coding is an artefact of where the investigator-chosen boundary falls, since the eight-or-more-day category collapses a graded range (0.31 at day 8 rising to 0.47 by day 45) into a single value of 0.39. The categorical coding is retained in Table 3 for comparability with the published no-show literature but should be read as a coarse summary of a continuous gradient (Figure 3). Individual coefficients on the lead-time spline basis are highly collinear with one another and individually imprecise, so inference rests on the joint test and the fitted curve rather than on single basis coefficients.

Splining age and lead time jointly changed the description of those two variables substantially but left every other adjusted association intact: female 0.945 to 0.937, emergency 0.173 to 0.176, free session 1.186 to 1.177, follow-up visit 0.261 to 0.260, psychiatrist 0.560 to 0.556, coach 0.447 to 0.433, device Unknown 1.053 to 1.064, and log (booking sequence) 0.996 to 0.997. The flexible specification therefore corrects the description of age and lead time without disturbing the rest of the model.

### 3.4. Cause-Specific Decomposition of Cancellation by Recorded Initiator

Because the primary outcome pools cancellations recorded with different initiators, the cancelled_by field was used to define a four-level outcome. In the de-duplicated regression subsample (n = 36,342), 27,049 bookings (74.4%) were completed, 4330 (11.9%) were cancelled by the treating provider, 3551 (9.8%) were cancelled by the user, and 1412 (3.9%) were cancelled by an administrator or had no recorded initiator. Provider-initiated cancellations therefore account for 4330 of the 9293 cancellations in this subsample (46.6%)—nearly half of the primary outcome. Three cause-specific binary logistic models were fitted, each comparing one cancellation type against completed bookings only, with the identical covariate set and user-level cluster-robust standard errors. Results are given in Table 2. A four-level multinomial model was also attempted; with the full covariate set it is not identified, because three session-type categories (package, emergency and supported) contain no provider-initiated cancellations at all and a fourth (free-AI) contains one, and the optimiser lands on materially different coefficient vectors for near-identical log-likelihoods. After pooling those four sparse session types, the multinomial converges cleanly and agrees closely with the separate binary models on every shared non-sparse term, so the cause-specific binary models are presented as the primary decomposition and the pooled multinomial as supplementary confirmation.

The decomposition changes the interpretation of the two largest associations in Table 3. For lead time, the two causes run in opposite directions. Relative to same-day booking, a lead time of eight or more days was associated with substantially lower odds of user-initiated cancellation (aOR 0.62, 95% CI 0.49–0.78, *p* < 0.001) and with far higher odds of provider-initiated cancellation (aOR 4.75, 95% CI 4.23–5.35, *p* < 0.001). Crude rates show the same reversal: as lead time rises from same-day to eight or more days, the user-initiated cancellation rate falls from 13.0% to 5.2% while the provider-initiated rate rises from 9.9% to 27.9%. The pooled adjusted odds ratio of 2.10 is therefore a weighted average of a protective user-side association and a strongly adverse provider-side one, and it should not be read as evidence that users abandon appointments they booked far in advance.

For provider specialty the contrast is equally sharp. In the provider-initiated model every specialty had markedly lower odds than psychologists (psychiatrists aOR 0.36, 95% CI 0.31–0.41; social/family counsellors 0.35, 0.31–0.40; family GPs 0.25, 0.19–0.33; coaches 0.16, 0.10–0.25; all *p* < 10^−13^). In the user-initiated model the same contrasts were 0.79 to 1.36 and mostly not statistically reliable (psychiatrists 0.88, 0.77–1.02, *p* = 0.089; social/family counsellors 0.94, 0.83–1.05, *p* = 0.273; coaches 0.79, 0.54–1.15, *p* = 0.213), and the one significant term—family GPs, 1.36 (1.13–1.64), *p* = 0.001—runs in the opposite direction to the pooled estimate. Crude rates make the same point: provider-initiated cancellation ranged from 3.7% among psychiatrists to 15.5% among psychologists, whereas the corresponding user-initiated rates all lay between 7.1% and 13.5%. The provider-specialty column of Table 3 therefore describes which specialties cancel on their patients, not which specialties’ patients cancel. Because the specialty signal is confined to provider-initiated cancellation and the extract contains no de-identified provider key, these differences may substantially reflect clustering of cancellation practice within individual providers rather than any property of the specialty itself (Section 4.4).

Four further associations are clarified by the decomposition. Free sessions, which carry higher pooled odds than standard paid sessions (aOR 1.19), showed no elevation at all in user-initiated cancellation (aOR 0.99, 95% CI 0.88–1.10, *p* = 0.814) and a clear elevation in provider-initiated cancellation (aOR 1.26, 95% CI 1.15–1.38, *p* < 0.001): free-session users are no more likely than paying users to cancel their own bookings, but their bookings are more likely to be cancelled by the provider. Unknown device type, null in the pooled model after de-duplication, is strongly and negatively associated with user-initiated cancellation (aOR 0.07, 95% CI 0.05–0.10) and positively associated with provider-initiated (aOR 1.37, 95% CI 1.21–1.56) and administratively recorded cancellation (aOR 2.72, 95% CI 2.19–3.37); among Unknown-device bookings, 1133 cancellations were provider-initiated against 42 user-initiated, confirming that Unknown marks a platform-side record state rather than a user attribute. Emergency bookings had no provider-initiated and exactly one user-initiated cancellation but significantly raised adjusted odds of falling into the administrative or unrecorded category (aOR 2.36, 95% CI 1.61–3.47, *p* < 0.001), which is examined further in Section 3.7. Finally, follow-up visits remained protective under both causes (user-initiated aOR 0.24, 95% CI 0.19–0.30; provider-initiated 0.28, 0.21–0.37), and the age association was entirely user-side (per decade: user-initiated aOR 0.76, 95% CI 0.71–0.81, *p* < 0.001; provider-initiated 0.98, 0.94–1.03, *p* = 0.434). Age is therefore a genuine user-behaviour signal, and a cleaner one than the pooled model conveys: younger users cancel their own appointments substantially more often, and this is not confounded by provider cancellation behaviour.

### 3.5. Prior Attendance and Cancellation History

Prior attendance and cancellation history are derivable from this extract, and they were derived using only information that had already resolved before the index booking was created (Section 2.5). In the de-duplicated regression subsample, 11,625 bookings (32.0%) had at least one prior booking whose outcome had resolved by the time the index booking was placed; the immediately preceding resolved booking had been completed for 10,032 bookings and cancelled for 1593, and 24,717 bookings had no resolved predecessor. Crude cancellation was 10.0% (1003/10,032) where the preceding resolved booking had been completed, 35.3% (562/1593) where it had been cancelled, and 31.3% (7728/24,717) where there was no resolved predecessor—a non-monotonic pattern in which having successfully attended, rather than merely having booked before, is what distinguishes the low-risk group.

Adding these covariates improved fit substantially. Relative to the primary model (log-likelihood −18,902.6; AIC 37,847.3; apparent AUC 0.700), a model adding the outcome of the preceding resolved booking and the number of prior cancellations gave log-likelihood −18,589.8, AIC 37,227.6 and apparent AUC 0.714. Cluster-robust joint Wald tests that the history terms are all zero gave χ^2^ = 208.71, df = 2, *p* = 4.8 × 10^−46^ for the preceding-outcome specification and χ^2^ = 275.27, df = 3, *p* = 2.2 × 10^−59^ for the count-based specification. What protects is prior successful attendance: a completed preceding booking carried aOR 0.39 (95% CI 0.33–0.47, *p* < 0.001) versus no resolved predecessor. Conditional on that, each additional prior cancellation raised the odds (aOR 1.39, 95% CI 1.28–1.50, *p* < 0.001), and in an alternative specification the proportion of prior bookings cancelled was a strong graded predictor (aOR 3.27, 95% CI 2.49–4.28, *p* < 0.001). Leakage was checked rather than assumed, comparing the leakage-free definition with a deliberately leakage-prone variant in which every earlier booking contributed regardless of when its outcome became known, holding the specification identical on both sides (the primary model plus the preceding-booking outcome, without the prior-cancellation count). A cancelled preceding booking was a significant risk factor under both definitions, but leakage inflated it roughly 2.4-fold: aOR 1.45 (95% CI 1.20–1.75, *p* < 0.001) leakage-free versus 3.47 (2.40–5.01, *p* < 0.001) leakage-prone, because users who cancel several bookings in one action generate apparent prediction from cancellations that had not yet occurred at booking time. The association therefore does not disappear once leakage is removed; it is smaller. The leakage-free definition is reported throughout, and the value of 0.94 (0.76–1.15, *p* = 0.541) obtained for a cancelled predecessor in the count-adjusted specification reported above reflects the number of prior cancellations absorbing that signal when both terms are entered together, not an absence of association.

Including prior history attenuated two of the coefficients highlighted in the previous version. The follow-up-visit association moved from aOR 0.26 (0.21–0.32) to 0.49 (0.41–0.57), a 46% reduction on the log-odds scale, so roughly half of what the pooled model attributes to an established care relationship is in fact prior attendance behaviour; it nevertheless remains substantively and statistically protective. The free-session association moved from 1.19 (1.10–1.27) to 1.08 (1.01–1.16), a 53% reduction on the log-odds scale. The new-provider-visit coefficient, null in the primary model (0.99, 0.83–1.18), became significantly adverse once history was adjusted (1.30, 1.13–1.49, *p* < 0.001), which is a new finding rather than a robustness footnote. Lead time (eight or more days: 2.10 to 2.22) and emergency sessions (0.173 to 0.175) were essentially unaffected, indicating that those associations are not confounded by unmeasured prior history. Visit type cannot substitute for history: among follow-up visits, 8133 followed a completed booking, 691 followed a cancellation, and 1601 had no resolved predecessor at booking time—three groups that the visit-type variable cannot distinguish. The residual limitation is left truncation: a user’s first observed booking may not be their first ever booking, although the platform launches shortly before the extract window opens, so the amount of unobserved history is likely to be small.

### 3.6. Calendar Time and Temporal Trend

**Figure 5:**
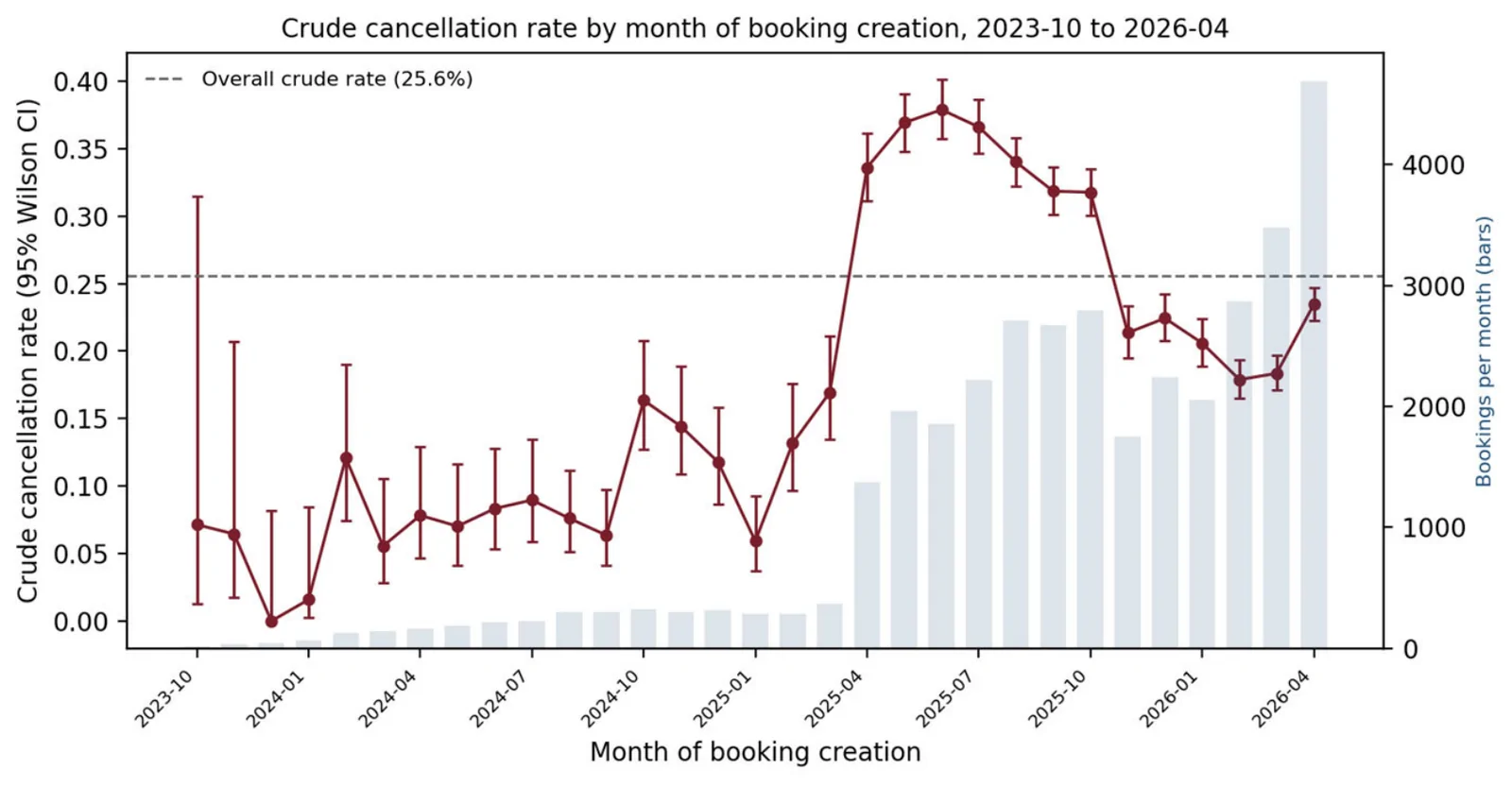
Monthly crude cancellation rate with Wilson 95% confidence intervals, and monthly booking volume, over the 31-month extract window (de-duplicated regression subsample: n = 36,342 bookings).

Calendar period, weekday of the scheduled session and scheduled time of day are all derivable from this extract; the only genuinely unavailable temporal field is the clock time at which a booking was placed, because the booking-creation timestamp is truncated to the day in the source query. The crude cancellation rate was far from stationary over the 31-month window. By calendar year of booking creation, it was 3.4% in 2023 (n = 88), 9.9% in 2024 (n = 2658), 31.0% in 2025 (n = 20,506) and 20.4% in 2026 (n = 13,090). By quarter it rose from 3.4% in 2023 Q4 to a peak of 36.4% in 2025 Q2, then declined to 18.7% in 2026 Q1 before rising to 23.5% in 2026 Q2. Monthly rates ranged from 0.0% (December 2023, n = 43) to 37.9% (June 2025, n = 1853), with an ordinary-least-squares trend across the 31 monthly rates of +0.0090 per month (*p* = 5.5 × 10^−6^, r = 0.718). Booking volume grew from 88 bookings in 2023 Q4 to 8398 in 2026 Q1, so the earliest periods are imprecise and 2023 is a poor reference year. The monthly series, with Wilson 95% confidence intervals and monthly booking volumes, is shown in Figure 5.

Calendar time is the single strongest covariate block added in any analysis reported here. A smooth restricted-cubic-spline trend in months since the start of the window gave a cluster-robust joint Wald statistic of χ^2^ = 251.34 on 4 degrees of freedom (*p* = 3.4 × 10^−53^) and raised the apparent AUC from 0.700 to 0.717. A categorical calendar-year block gave χ^2^ = 153.25, df = 3, *p* = 5.2 × 10^−33^, and the weekday of the scheduled session gave χ^2^ = 29.38, df = 6, *p* = 5.1 × 10^−5^ (Saturday aOR 1.15, 95% CI 1.05–1.26 versus Sunday; Wednesday 0.90, 0.82–0.99). Crude rates by weekday of the scheduled session ranged from 23.3% on Wednesday to 27.3% on Saturday, and by scheduled hour from 15.4% at midnight to 35.6% at 09:00.

Most associations in Table 3 are robust to calendar adjustment: lead time of eight or more days moved from 2.10 to 2.06, age from 0.896 to 0.897, gender remained null, follow-up visit moved from 0.26 to 0.29, emergency from 0.173 to 0.201, and provider specialty attenuated modestly but remained strongly significant (psychiatrists 0.56 to 0.60, coaches 0.45 to 0.52). Two do not. The free-AI association attenuates by roughly two-thirds on the log-odds scale, from aOR 0.58 (0.49–0.69) to 0.82 (0.68–0.99) with a smooth calendar trend, and to 0.83 (0.69–1.01), no longer statistically reliable, once weekday is also included. Free-AI sessions were introduced at a particular point in the platform’s history, so their apparently protective association is largely a period effect; we therefore do not report it as a finding. The free-session association moves in the opposite direction and strengthens, from 1.19 (1.10–1.27) to 1.49 (1.36–1.64). We report the smooth calendar-time model as a pre-specified sensitivity analysis rather than folding it into the primary model, so that Table 3 remains directly comparable with the published literature. The non-stationarity of the outcome is nevertheless a substantial threat to the internal validity of pooled estimates over this window and is recorded as such in Section 4.4.

### 3.7. Sensitivity Analyses: Alternative Outcome Definitions

Two alternative outcome definitions were examined, both on the de-duplicated regression subsample. First, the regression was re-estimated using the more restrictive was_cancelled flag as the outcome (7923 flagged bookings, 21.8% of 36,342; every unflagged booking, including the 1388 status_label-cancelled records that carry no cancellation row, treated as a control; apparent AUC 0.733). Every headline association remained in the same direction, and most associations strengthened slightly: follow-up visit aOR 0.23 (95% CI 0.19–0.29), lead time of eight or more days 2.61 (2.35–2.90), lead time 4–7 days 1.39 (1.27–1.53), age 0.89 (0.86–0.93) per decade, psychiatrist 0.58 (0.52–0.64), coach 0.34 (0.25–0.46) and free session 1.09 (1.02–1.18); recorded female gender became significant (0.90, 0.84–0.97) and device Unknown protective (0.78, 0.71–0.87), in both cases for the same reason as under the concordant definition described next. The emergency term collapses to a boundary-like value under this definition (aOR 0.003, 95% CI 0.000–0.018) because exactly one emergency booking carries the flag. So that no coefficient from this model is reported selectively, the remaining estimates under the was_cancelled-flag definition are the following: device iOS 1.05 (0.96–1.15), social/family counsellor 0.57 (0.52–0.62), family GP 0.55 (0.47–0.65), package 0.009 (0.002–0.047), free-AI 0.38 (0.31–0.47), supported 0.67 (0.42–1.06), new-provider visit 0.99 (0.82–1.19), lead time 1 day 0.89 (0.82–0.96), lead time 2–3 days 1.03 (0.94–1.13), and log (booking sequence) 0.99 (0.86–1.13). Second, a strictly concordant definition was used in which cases were bookings that were both status_label-cancelled and flagged (n = 7905) and controls were bookings that were both status_label-completed and unflagged (n = 27,031); all 1406 discordant records in the regression subsample were excluded rather than recoded, leaving 34,936 bookings from 20,934 users, 22.6% events, and an apparent AUC of 0.733. The headline associations held under this stricter definition: follow-up visit aOR 0.23 (95% CI 0.19–0.28), lead time of eight or more days 2.53 (2.27–2.81), lead time 4–7 days 1.37 (1.25–1.51), age 0.89 (0.86–0.93) per decade, psychiatrist 0.56 (0.51–0.63), coach 0.34 (0.25–0.47) and free session 1.12 (1.04–1.20). Recorded female gender, null under the primary definition, reached significance under the concordant definition (aOR 0.91, 95% CI 0.85–0.98, *p* = 0.015). Device Unknown became protective under this definition (0.83, 0.74–0.92), which is consistent with Unknown marking a record state whose bookings disproportionately lack an explicit cancellation row. So that no coefficient from this model is reported selectively either, the remaining estimates under the concordant definition are the following: device iOS 1.04 (0.95–1.13), device Unknown 0.83 (0.74–0.92), social/family counsellor 0.56 (0.52–0.62), family GP 0.61 (0.52–0.72), package 0.011 (0.002–0.052), free-AI 0.39 (0.32–0.48), supported 0.69 (0.43–1.11), new-provider visit 0.99 (0.82–1.19), lead time 1 day 0.88 (0.81–0.95), lead time 2–3 days 1.02 (0.93–1.11), and log (booking sequence) 0.99 (0.86–1.13); the emergency estimate under this definition is discussed below. The full table on the log-odds scale, with standard errors, is available from the corresponding author on request.

Neither alternative definition resolves the emergency-session estimate, and neither can, because the problem is a single event rather than the treatment of discordant records. Among the 1135 emergency bookings in the regression subsample, there were no provider-initiated and one user-initiated cancellation. Their 72 recorded cancellations were almost entirely administrative status changes with no cancellation record and no recorded initiator (71 of 72), and emergency bookings had significantly raised adjusted odds of falling into that administrative category (aOR 2.36, 95% CI 1.61–3.47). Under the concordant definition 1064 emergency bookings remain, of which exactly one is a case; the resulting coefficient is driven to a boundary-like value with an interval spanning nearly two orders of magnitude (aOR 0.003, 95% CI 0.000–0.018), and Firth penalisation returns a finite but equally uninterpretable value. This is quasi-complete separation, not an estimate. We therefore regard the adjusted odds ratio of 0.17 obtained under the primary definition as reflecting how emergency bookings are coded by the platform rather than any behavioural effect of urgency, and we have removed the inference that perceived urgency drives adherence from the Abstract, Discussion and Conclusions.

### 3.8. Covariate Overlap and Sparsity

Covariate overlap was examined directly, because conventional collinearity measures do not capture empty and near-empty cells. Of the 30 session types by provider-specialty combinations, 5 are empty and 3 more contain fewer than 20 bookings. The dependence is concentrated in one category: free-AI sessions are delivered almost exclusively by psychiatrists (95.3%) and coaches (4.7%), so three of the five specialty cells in that row are structurally empty. Session type by lead-time category has no empty cells but 7 cells with fewer than 20 bookings, all in the free-AI, emergency and supported rows; emergency and free-AI bookings are 94.5% and 91.7% same-day respectively, so those coefficients are estimated with almost no within-category lead-time variation. Across the full three-way cross-classification of session type, provider specialty and lead-time category, 115 of 150 possible cells (76.7%) are non-empty; 51 of those (44.3%) contain fewer than 20 bookings but jointly cover only 343 bookings, 0.94% of the sample, while 43 cells with at least 100 bookings cover 35,056 bookings, 96.5% of the sample. Variance-inflation factors are all low (maximum 3.378, for follow-up visit), and generalised variance-inflation factors by covariate block are all far below the equivalent of a VIF of 5 (largest GVIF^(1/2df) = 1.78, for log (booking sequence)). As anticipated, the collinearity diagnostics do not detect the problem, because the problem is sparse cells rather than correlated predictors; both are therefore reported.

The consequences for the estimates are limited. Excluding the 2386 free-AI and 1135 emergency bookings, the two categories with the most structural dependence, changed lead time of eight or more days from 2.100 to 2.105, psychiatrist from 0.560 to 0.554, follow-up visit from 0.261 to 0.255, and age from 0.896 to 0.892; restricting the sample to standard, free and package sessions gave 2.118, 0.555, 0.252 and 0.891 respectively. The largest movement anywhere was for coaches (0.447 to 0.417), well within its confidence interval. The structural dependence is therefore real and is documented here, but it does not destabilise the estimates for the covariates that carry the paper’s conclusions; the coefficients that should be read with caution are those for free-AI, emergency and supported sessions.

### 3.9. Exploratory Model Fit

As an exploratory descriptive summary, the apparent AUC of the fitted primary model in the analysis sample was 0.700: the model assigns a higher predicted probability of cancellation to a randomly selected cancelled booking than to a randomly selected completed booking in approximately 70% of such pairs. This value describes fit in the data used to estimate the model. Because no internal, temporal, or external validation was performed, it should not be read as an estimate of performance in future bookings or on other platforms, and the model is not proposed for operational use. For context, adding the leakage-free prior-history covariates raises the apparent AUC to 0.714 and adding the smooth calendar-time trend raises it to 0.717, so most of the discriminatory information the covariate set omits is prior attendance behaviour and secular trend rather than non-linear structure among the covariates already included. An exploratory gradient-boosted-tree comparison reported in the previous version has been removed from this revision: its only substantive content was a small difference in discrimination relative to this logistic model, which the prior-history and calendar-time results above show to be attributable to information the logistic model was not given, and feature-importance rankings from a fitted algorithm cannot corroborate a covariate-adjusted association in any case.

## 4. Discussion

### 4.1. Principal Findings

On a Saudi commercial digital mental health platform, cancellation of a booked session was most strongly associated with operational features of the booking—visit type, lead time, session type, and provider specialty—and only weakly with user demographics. The central result of the present analysis, however, is that most of those associations describe provider-side scheduling behaviour rather than patient non-adherence. Provider-initiated cancellations account for 46.6% of the primary outcome, and decomposing cancellation by recorded initiator reverses or nullifies the two largest associations. A lead time of eight or more days doubles pooled cancellation odds, but it lowers the odds that a user cancels their own booking (aOR 0.62) and quadruples the odds that the provider cancels it (aOR 4.75). Free sessions carry 19% higher pooled odds than standard paid sessions but show no elevation at all in user-initiated cancellation (aOR 0.99) and a clear elevation in provider-initiated cancellation (aOR 1.26). Provider-specialty differences of 0.16 to 0.36 versus psychologists in the provider-initiated model shrink to 0.79 to 1.36, mostly null, in the user-initiated model. Because that signal is confined to provider-initiated cancellation and the extract contains no de-identified individual provider key, these specialty differences may substantially reflect the clustering of cancellation practice within a small number of individual providers rather than any property of the specialty (Section 4.4). Two associations survive the decomposition as genuine user-behaviour signals, and are strengthened by it: age, whose association is entirely user-side (aOR 0.76 per decade for user-initiated cancellation versus 0.98 for provider-initiated) and concentrated between 18 and about 25 years rather than spread evenly across the age range; and follow-up visits with an established provider, protective under both causes, although roughly half of that association is prior attendance behaviour rather than the care relationship itself once leakage-free prior history is adjusted (aOR 0.26 to 0.49). Prior attendance history is itself a strong predictor in these data: a completed preceding booking carries aOR 0.39, and each additional prior cancellation aOR 1.39. Recorded gender carried no adjusted association, and the booking sequence number, independent of visit type, was uninformative. The very low adjusted odds for emergency sessions are artefactual: emergency bookings are essentially never cancelled through the platform’s explicit cancellation pathway, and their recorded cancellations are administrative status changes, so the previous inference that perceived urgency drives adherence is withdrawn. The covariates jointly separated cancelled from completed bookings only moderately (apparent AUC 0.70), consistent with the explanatory rather than predictive aim of the analysis.

### 4.2. Comparison with Existing Literature

Two patterns deserve comment in relation to the broader no-show literature, and in both cases the cause-specific decomposition changes what can legitimately be claimed. First, the pooled lead-time gradient observed here (eight or more days doubling cancellation odds versus same-day booking) is directionally consistent with the central conclusion of the Dantas et al. systematic review of 105 no-show studies, which identified lead time as one of the two most consistent determinants of non-attendance across specialties [10], a finding echoed in a primary-care-specific systematic review of missed appointments [20]. In the previous version we attributed this gradient to forgetting and to changes in motivational state between booking and session date. That mechanism is contradicted by the present data: users are markedly less likely to cancel their own long-lead bookings (aOR 0.62 at eight or more days), and the pooled gradient is generated almost entirely by provider-initiated cancellation (aOR 4.75). The comparison with the clinic-based no-show literature is therefore not like-for-like, because that literature largely concerns patient non-attendance whereas the dominant component of the association here is provider-side schedule instability at long horizons. The spline analysis also removes the threshold reading: cancellation risk is flat over the first three days after booking and then rises smoothly to roughly three to four weeks of lead time, with no discontinuity at eight days, so the apparent threshold reported previously was an artefact of where the investigator-chosen category boundary fell.

Second, the finding that follow-up visits show lower cancellation than first visits—a 74% reduction in adjusted odds on the de-duplicated sample—aligns in direction with the international finding that established care relationships, continuity of care, and therapeutic alliance reduce missed appointments [11,21,22,23]. On a digital commercial platform, where there is no compulsion to maintain continuity of care with a single provider, the magnitude is striking. It must nonetheless be discounted. Follow-up status is by construction selected for prior engagement, and once leakage-free prior attendance history is adjusted, the association attenuates by 46% on the log-odds scale, to aOR 0.49. What protects is having successfully attended before (aOR 0.39 for a completed preceding booking), not merely having booked before; and among follow-up visits, 8133 followed a completed booking while 691 followed a cancellation, groups that visit type alone cannot distinguish. Retention into a second session therefore remains a potentially important transition point to be examined prospectively rather than a demonstrated operational mechanism, and roughly half of what we previously attributed to the care relationship is more parsimoniously described as prior attendance behaviour. Consistent with this, the new-provider-visit coefficient becomes significantly adverse once history is adjusted (aOR 1.30, 95% CI 1.13–1.49), suggesting that switching providers after a prior booking carries elevated rather than neutral risk.

A finding that diverges from the broader literature is the absence of a gender effect. Population-based no-show studies often report male gender as a risk factor for missed appointments, though effects are typically small and inconsistent across settings. On the present platform, after adjustment, the female-versus-male odds ratio was close to unity (0.95, *p* = 0.119), and it remained null in both the user-initiated (0.92, 95% CI 0.83–1.03) and provider-initiated (0.92, 0.85–1.00) cause-specific models, so the null result is not an artefact of pooling. It does reach conventional significance under the stricter concordant outcome definition (0.91, 0.85–0.98), which we report but do not emphasise. Two explanations are plausible: (i) the platform user population is over two-thirds female and may not contain the male-specific avoidance patterns documented in mixed-population samples, and (ii) gender effects in non-platform settings may operate through transport and time-cost barriers that are absent in fully online care.

A third finding worth situating is the differential by session type, particularly the higher cancellation rate among free sessions. In the previous version we interpreted this through behavioural-economic accounts of appointment commitment, including the disproportionate weight consumers place on a zero price relative to any small positive price [21,24]. That interpretation is not supported by the present analysis and is withdrawn. Free-session users are no more likely than paying users to cancel their own bookings (user-initiated aOR 0.99, 95% CI 0.88–1.10, *p* = 0.814); what distinguishes free sessions is that providers cancel them more often (provider-initiated aOR 1.26, 95% CI 1.15–1.38), and the pooled association also attenuates by roughly half on the log-odds scale once prior attendance history is adjusted (1.19 to 1.08). A commitment-based account of the free-session differential would require evidence of elevated user-initiated cancellation, and that evidence is absent. The pattern is better read as an operational one: free-tier bookings, used predominantly for acquisition and first-touch encounters, appear to receive less stable provider scheduling. The reduction in cancellation among emergency sessions reported previously (aOR 0.18) likewise no longer supports any inference about urgency and adherence, since emergency bookings carry no provider-initiated and one user-initiated cancellation and their recorded cancellations are administrative in nature; that sentence has been removed. Finally, the apparently protective association for free-AI sessions is largely a period effect that does not survive adjustment for calendar time and is not advanced here as a finding.

### 4.3. Implications for Platform Operations

Three hypotheses for operational intervention follow from these associations; each would require prospective evaluation—for example, a randomised trial or platform-level A/B test—before implementation, and the cause-specific decomposition changes which actor each is aimed at. First, the lead-time gradient is an actionable lever, but a provider-side one. Because long-lead bookings are cancelled predominantly by providers rather than by users, interventions directed at users—capping advance booking, intensifying reminders for long-lead bookings, or prompting users to reschedule near the session date—address the wrong actor, and capping advance booking would in addition restrict scheduling flexibility for users who need it. The corresponding provider-side measures are the hypotheses these data support: improving the stability of provider availability at horizons beyond one week, limiting how far ahead a provider’s calendar can be opened relative to how reliably it is honoured, requiring earlier confirmation of long-lead slots, and monitoring provider-level cancellation as an operational quality indicator. Second, the first-to-second-session transition remains a candidate high-leverage point in the user journey, but it should be framed around prior attendance rather than around visit labelling. The strongest user-side signal in these data is whether a user has previously completed a session, so interventions that secure a first completed attendance, and that identify users whose prior bookings have been cancelled—whose risk rises with each additional prior cancellation—are the more direct target. Interventions premised on converting free sessions to paid sessions are not supported, because free-session users are not the ones cancelling. Third, the two demographic variables available here carried limited incremental information after adjustment for booking characteristics, so targeting on recorded gender and age alone is unlikely to be an efficient allocation of effort on this platform. That is a claim about these two variables only; it is not a claim that demographic, socioeconomic, or equity-based targeting is uninformative in general, which this extract cannot assess (Section 4.4). The one demographic signal worth prospective attention is that the age gradient is genuinely user-side and concentrated in users aged approximately 18 to 25 years.

### 4.4. Limitations

Several limitations should be considered. The data come from a single commercial platform in Saudi Arabia, and external validity to public-sector services, to other Gulf platforms, or to non-Arabic-language settings is unconfirmed. A data artefact identified during the present revision should be recorded first, because it affected two previously reported estimates. The anonymised booking identifier is not unique in the source extract: because the extraction query left-joins a separate cancellation table and the row-numbering window functions are evaluated after that join, a booking with more than one cancellation record was emitted once per record and each additional emission was relabelled as a follow-up visit (Section 2.3). Three hundred and thirty-one rows, 0.90% of the previously reported analytic frame, were affected, but they were highly concentrated: they made up 20.5% of the “follow-up visit and cancelled” cell and 13.0% of the “device Unknown and cancelled” cell. After de-duplication, the previously reported 26% elevation in cancellation odds for Unknown device type is null and is withdrawn, the follow-up-visit association strengthens from 0.35 to 0.26, and the family-GP contrast is no longer statistically reliable. Model fit improves. We report this in full rather than silently correcting it, and we note that user-level cluster-robust standard errors absorb part but not all of the problem, since all emissions of one booking fall inside a single user cluster while the point estimates remain biased. The cancellation outcome combines provider-initiated, user-initiated, administratively recorded cancellations, and probable no-shows resolved at the platform level. This heterogeneity is now addressed directly rather than deferred: Section 3.4 reports cause-specific models by recorded initiator, and they show that several pooled associations are properties of provider behaviour rather than user behaviour. A fully identified four-level multinomial model could not be fitted with the complete covariate set, because three session-type categories contain no provider-initiated cancellations at all; the multinomial is therefore reported only after pooling the sparse session types, and the cause-specific binary models carry the inference. No-shows still cannot be separated from administrative cancellation, because the extract does not distinguish them: the 1404 bookings marked cancelled without an explicit cancellation row are exactly those with no recorded initiator, and their true nature cannot be determined from the available fields. Standard errors were clustered at the user level only. The extract links each booking to a provider specialty but not to an individual, de-identified provider code, so provider-level clustering, provider fixed effects, and multiway clustering by user and provider could not be implemented. This limitation is more consequential than it appeared in the previous version: provider-initiated cancellations are 46.6% of the primary outcome, and the provider-specialty associations are essentially confined to the provider-initiated model, so the observed specialty differences may substantially reflect the clustering of cancellation practice within a small number of individual providers rather than any property of the specialty. Every statement about provider specialty in this paper should be read with that caution, and this should be revisited if a future extract includes a de-identified provider key. The crude cancellation rate is not stationary over the study window: it ranges from 3.4% in 2023 to 31.0% in 2025 and 20.4% in 2026, monthly rates range from 0.0% to 37.9%, and calendar time is the strongest single covariate block we examined (Section 3.6). We do not know which platform changes produced this trajectory, and the pooled estimates should be read as averages over a non-stationary period rather than as stable parameters. One specific association, the apparently protective effect of free-AI sessions, is largely a period effect and is not reported as a finding. Prior cancellation and attendance history, which the previous version stated was not available in the extract, is in fact derivable, and that statement was incorrect. It has been derived using only information resolved before the index booking, and it is reported in Section 3.5. The residual limitation is left truncation: a user’s first observed booking may not be their first ever booking, so history is censored at the start of the extract window, although the platform launches shortly before that window opens. A closely related correction applies to calendar and scheduling variables. The previous version stated that day of week, time of day, and calendar period were not available in the extract; this was also incorrect. Calendar year, quarter and month of booking creation, weekday of the scheduled session, and scheduled time of day are all derivable, and all are now reported (Section 3.6). The only genuinely unavailable temporal field is the clock time at which a booking was placed, because the booking-creation timestamp is truncated to the day in the source query. Price paid, promotional discounts, payment completion, reminder delivery, cancellation policy, and documented platform changes to the interface, reminder system, or provider network remain unavailable and unmodelled. Recorded age could not be validated against a date of birth, because the extract contains no date-of-birth field; verification was limited to range checking, implausible values were quantified, and ages above 90 years were excluded from the primary model (Section 2.3 and Section 2.5). Several constructs not available in the platform extract—symptom severity at booking, prior treatment history outside the platform, financial pressure—are likely confounders of the observed associations, particularly the session-type and follow-up effects; more generally, users are not randomly assigned to session type, provider, or follow-up status, so self-selection into free, package, or emergency sessions, and into continued care with the same provider, likely contributes to the associations reported here alongside any causal effect of the booking feature itself. Structural overlap among covariates is documented in Section 3.8: five of thirty session-type by specialty cells are empty, free-AI sessions are delivered almost exclusively by psychiatrists, and emergency and free-AI bookings carry almost no lead-time variation, so the coefficients for those three session types should be read with particular caution even though the estimates for the covariates carrying this paper’s conclusions are stable to their exclusion. Unadjusted hypothesis tests have been removed from Table 1, because observations are not independent within users and nominal *p*-values would be anticonservative. The negative-lead-time exclusion (n = 970, 2.1%) was treated as a data anomaly. The apparent AUC of 0.70 indicates modest-to-moderate separation of cancelled from completed bookings: the fitted model assigns a higher predicted risk to a randomly selected cancelled booking than to a randomly selected completed booking in approximately 70% of such pairs. This is a development-sample value; no internal, temporal, or external validation was performed, no prediction model is proposed, and generalisability to future bookings and to other platforms is unknown. Because the dataset lacks a within-platform device fingerprint or verified national identifier, we cannot rule out a small number of duplicate user accounts (the same person registering more than once), which would understate the true number of unique users and could inflate apparent user-level clustering. The available demographic information is limited to age and a binary recorded gender field; socioeconomic status, employment, education, region, disability, digital literacy, and financial constraints were not captured and could not be examined, so the modest role of demographic variables reported here should not be read as evidence that demographic targeting in general is uninformative, only that the two demographic variables available in this extract carried limited incremental information after adjustment for booking characteristics. Recorded gender in this dataset reflects only the two values present in the registration field (“Female”/”Male”) rather than a comprehensive assessment of gender identity; we cannot assess how non-binary or gender-diverse users are represented in, or excluded from, this field, and digital mental health access and engagement may differ meaningfully in these populations. Free-AI sessions (Section 2.1) are markedly shorter (median 5 min) and almost always same-day, unlike standard professional sessions; although they were retained in the primary model because they are booked, staffed, and cancelled through the same platform pipeline, their direct comparability to full-length consultations is limited, and estimates for this category should be interpreted with that difference in mind.

## 5. Conclusions

On a Saudi commercial digital mental health platform, session cancellation was associated most strongly with the operational features of the booking—visit type, lead time, session type, and provider specialty—while the available demographic variables (age and recorded gender) contributed limited incremental information after adjustment. The principal contribution of this analysis is to show that these pooled associations should not be read as measures of patient adherence. Provider-initiated cancellations are 46.6% of the recorded outcome, and once cancellation is decomposed by recorded initiator, the lead-time gradient reverses direction on the user side, the free-session differential disappears on the user side, and provider-specialty differences are confined almost entirely to provider-initiated cancellation. Operational attention is therefore better directed at the stability of provider schedules at long booking horizons than at user reminders or pricing. The associations that do describe user behaviour are the age gradient, which is entirely user-side and concentrated in users aged approximately 18 to 25 years, and prior attendance history, which is derivable from routinely collected booking records and is a strong graded predictor: having previously completed a session is protective, and risk rises with each prior cancellation. We make no inference that perceived urgency drives adherence; the very low adjusted odds observed for emergency sessions reflect how the platform records those bookings rather than user behaviour. These findings remain exploratory and hypothesis-generating, and the candidate intervention targets they identify should be evaluated prospectively before implementation. The analysis extends the international no-show literature into the commercial telemental health setting in the Gulf, illustrating why routinely collected cancellation outcomes should be decomposed by initiator before being interpreted as engagement measures.

## Figures and Tables

**Table 1:** Sample characteristics by cancellation status (de-duplicated analytic frame: n = 36,544 bookings, 21,904 users).

Variable	Total, n (%)	Completed (n = 27,196)	Cancelled (n = 9348)	Cancellation Rate, % (95% CI)
Female	25,247 (69.1)	18,800 (69.1)	6447 (69.0)	25.54 (25.00–26.08)
Male	11,297 (30.9)	8396 (30.9)	2901 (31.0)	25.68 (24.88–26.49)
Mean age, years (SD)	29.2 (8.9)	29.5 (8.8)	28.3 (8.9)	—
Device—iOS	26,944 (73.7)	20,414 (75.1)	6530 (69.9)	24.24 (23.73–24.75)
Android	4492 (12.3)	3340 (12.3)	1152 (12.3)	25.65 (24.39–26.94)
Unknown	4999 (13.7)	3358 (12.3)	1641 (17.6)	32.83 (31.54–34.14)
Web	109 (0.3)	84 (0.3)	25 (0.3)	22.94 (16.05–31.67)
Provider—Psychologist	23,378 (64.0)	16,690 (61.4)	6688 (71.5)	28.61 (28.03–29.19)
Psychiatrist	5994 (16.4)	5043 (18.5)	951 (10.2)	15.87 (14.96–16.81)
Social/Family Counsellor	5346 (14.6)	4134 (15.2)	1212 (13.0)	22.67 (21.57–23.81)
Family GP	1210 (3.3)	832 (3.1)	378 (4.0)	31.24 (28.69–33.91)
Coach	542 (1.5)	443 (1.6)	99 (1.1)	18.27 (15.24–21.74)
Speech Therapist	61 (0.2)	45 (0.2)	16 (0.2)	26.23 (16.84–38.44)
Family Consultant	13 (<0.1)	9 (<0.1)	4 (<0.1)	30.77 (12.68–57.63)
Session type—Normal	13,690 (37.5)	10,456 (38.4)	3234 (34.6)	23.62 (22.92–24.34)
Free	16,723 (45.8)	11,301 (41.6)	5422 (58.0)	32.42 (31.72–33.14)
Package	2397 (6.6)	2175 (8.0)	222 (2.4)	9.26 (8.17–10.49)
Free-AI	2403 (6.6)	2055 (7.6)	348 (3.7)	14.48 (13.13–15.95)
Emergency	1140 (3.1)	1068 (3.9)	72 (0.8)	6.32 (5.05–7.88)
Supported	170 (0.5)	131 (0.5)	39 (0.4)	22.94 (17.26–29.82)
Programme	21 (<0.1)	10 (<0.1)	11 (0.1)	52.38 (32.37–71.66)
Visit—First	21,489 (58.8)	14,548 (53.5)	6941 (74.3)	32.30 (31.68–32.93)
Follow-up	10,447 (28.6)	9292 (34.2)	1155 (12.4)	11.06 (10.47–11.67)
New provider	4608 (12.6)	3356 (12.3)	1252 (13.4)	27.17 (25.91–28.47)
Lead time—same day	19,495 (53.4)	14,837 (54.6)	4658 (49.8)	23.89 (23.30–24.50)
1 day	6004 (16.4)	4572 (16.8)	1432 (15.3)	23.85 (22.79–24.95)
2–3 days	4175 (11.4)	3074 (11.3)	1101 (11.8)	26.37 (25.06–27.73)
4–7 days	3419 (9.4)	2404 (8.8)	1015 (10.9)	29.69 (28.18–31.24)
≥8 days	3451 (9.4)	2309 (8.5)	1142 (12.2)	33.09 (31.54–34.68)

Percentages in the Total column are of all 36,544 bookings in the de-duplicated analytic frame; percentages in the Completed and Cancelled columns describe the composition of those two groups. The final column gives the crude cancellation rate within each category with a Wilson 95% confidence interval. Unadjusted hypothesis tests have been removed from this table: bookings are clustered within users (4233 of the 21,904 users contribute 18,873 of the 36,544 bookings, one user contributing 207), so nominal chi-square and *t*-test *p*-values ignore this dependence and are anticonservative, and within-category risk with an interval is more directly informative than a *p*-value at this sample size. Percentages may not sum to 100 due to rounding.

**Table 2:** Cause-specific adjusted odds ratios for booking cancellation by recorded initiator (de-duplicated regression subsample: n = 36,342 bookings, 21,758 users).

Covariate	Any Cancellation (Primary)	User-Initiated	Provider-Initiated	Administrative or Unrecorded
Female (vs. Male)	0.95 (0.88–1.01)	0.92 (0.83–1.03)	0.92 (0.85–1.00)	1.14 (0.98–1.31)
Device—iOS (vs. Android)	1.01 (0.93–1.10)	1.17 (1.03–1.32) *	0.91 (0.82–1.02)	0.84 (0.68–1.03)
Device—Unknown (vs. Android)	1.05 (0.95–1.16)	0.07 (0.05–0.10) *	1.37 (1.21–1.56) *	2.72 (2.19–3.37) *
Provider—Psychiatrist (vs. Psychologist)	0.56 (0.51–0.62) *	0.88 (0.77–1.02)	0.36 (0.31–0.41) *	0.55 (0.43–0.71) *
Provider—Social/Family Counsellor	0.61 (0.56–0.67) *	0.94 (0.83–1.05)	0.35 (0.31–0.40) *	0.93 (0.78–1.11)
Provider—Family GP	0.88 (0.77–1.01)	1.36 (1.13–1.64) *	0.25 (0.19–0.33) *	2.68 (2.17–3.30) *
Provider—Coach	0.45 (0.35–0.57) *	0.79 (0.54–1.15)	0.16 (0.10–0.25) *	0.89 (0.61–1.29)
Session—Free (vs. Standard)	1.19 (1.10–1.27) *	0.99 (0.88–1.10)	1.26 (1.15–1.38) *	1.90 (1.55–2.34) *
Session—Package	0.47 (0.36–0.63) *	0.03 (0.01–0.15) *	no events (separated)	7.33 (5.54–9.71) *
Session—Free-AI	0.58 (0.49–0.69) *	0.59 (0.47–0.75) *	1 event (unstable)	2.73 (1.96–3.82) *
Session—Emergency	0.17 (0.13–0.23) *	0.003 (0.000–0.024) *	no events (separated)	2.36 (1.61–3.47) *
Session—Supported	0.87 (0.56–1.36)	0.90 (0.57–1.42)	no events (separated)	2.97 (1.46–6.04) *
Follow-up visit (vs. First)	0.26 (0.21–0.32) *	0.24 (0.19–0.30) *	0.28 (0.21–0.37) *	0.54 (0.37–0.80) *
New-provider visit (vs. First)	0.99 (0.83–1.18)	1.22 (1.02–1.47) *	0.70 (0.54–0.92) *	1.04 (0.74–1.48)
Lead time of 1 day (vs. same day)	0.87 (0.81–0.94) *	0.92 (0.83–1.02)	0.86 (0.77–0.96) *	0.82 (0.69–0.97) *
Lead time of 2–3 days	0.98 (0.91–1.07)	0.79 (0.69–0.90) *	1.26 (1.13–1.41) *	0.80 (0.66–0.97) *
Lead time of 4–7 days	1.26 (1.15–1.38) *	0.76 (0.65–0.88) *	2.02 (1.81–2.26) *	0.77 (0.62–0.96) *
Lead time of ≥8 days	2.10 (1.90–2.33) *	0.62 (0.49–0.78) *	4.75 (4.23–5.35) *	0.78 (0.60–1.01)
Age, per 10 years	0.90 (0.86–0.93) *	0.76 (0.71–0.81) *	0.98 (0.94–1.03)	0.92 (0.85–0.99) *
log (booking sequence)	1.00 (0.88–1.13)	1.13 (1.01–1.28) *	0.76 (0.62–0.92) *	1.02 (0.85–1.23)

* *p* < 0.05. Values are adjusted odds ratios with 95% confidence intervals. Each cause-specific model compares one cancellation type against completed bookings only, using the identical covariate set and user-level cluster-robust standard errors. Model sizes: any cancellation 36,342 bookings (9293 events, 21,758 users); user-initiated 30,600 bookings (3551 events, 17,676 users); provider-initiated 31,379 bookings (4330 events, 19,405 users); administrative or unrecorded 28,461 bookings (1412 events, 16,916 users). Session-type categories containing no provider-initiated cancellations are marked accordingly; the free-AI estimate in that column rests on a single event and is not interpretable. Firth penalised logistic regression with profile penalised-likelihood confidence intervals was fitted for these separated terms and returned finite bounded values, but these remain uninterpretable because they rest on zero or one event, and Firth intervals are not cluster-robust; on every non-separated term the penalised and unpenalised estimates agree to three decimal places. All four columns were estimated on the regression subsample (36,342 bookings, 21,758 users) rather than on the age-restricted primary estimation sample used for Table 3 (36,337 bookings, 21,753 users), so that the four columns share one estimation frame; the two frames differ only by the 5 bookings with a recorded age above 90 years, and the “Any cancellation” column differs from Table 3 by at most 0.002.

**Table 3:** Adjusted odds ratios for booking cancellation (primary model, estimated on the primary estimation sample, i.e., the de-duplicated regression subsample restricted to recorded age 90 years or below: n = 36,337 bookings, 21,753 users).

Variable	aOR	95% CI	*p*
Female (vs. Male)	0.95	0.88–1.01	0.119
Age, per 10 years	0.90	0.86–0.93	<0.001
Device iOS (vs. Android)	1.01	0.93–1.10	0.841
Device Unknown	1.05	0.95–1.16	0.312
Provider—Psychiatrist (vs. Psychologist)	0.56	0.50–0.62	<0.001
Provider—Social/Family Counsellor	0.61	0.56–0.67	<0.001
Provider—Family GP	0.88	0.77–1.01	0.066
Provider—Coach	0.45	0.35–0.57	<0.001
Session—Free (vs. Standard)	1.19	1.10–1.27	<0.001
Session—Package	0.47	0.36–0.63	<0.001
Session—Free-AI	0.58	0.49–0.69	<0.001
Session—Emergency (unstable; see note)	0.17	0.13–0.23	<0.001
Session—Supported	0.87	0.56–1.36	0.548
Follow-up visit (vs. First)	0.26	0.21–0.32	<0.001
New-provider visit	0.99	0.83–1.18	0.900
Lead time 1 day (vs. same day)	0.87	0.81–0.94	<0.001
Lead time 2–3 days	0.98	0.90–1.07	0.708
Lead time 4–7 days	1.26	1.15–1.37	<0.001
Lead time ≥8 days	2.10	1.90–2.33	<0.001
log (booking sequence)	1.00	0.88–1.13	0.954

Logistic regression with cluster-robust standard errors at the user level, estimated on the primary estimation sample (Section 2.5). aOR = adjusted odds ratio; CI = confidence interval. Model pseudo-R^2^ (McFadden) = 0.085; apparent AUC = 0.700 (analysis sample; no validation performed). The emergency-session estimate is retained for completeness but is unstable and probably artefactual, because emergency bookings have essentially no cancellations recorded through the platform’s explicit cancellation pathway (Section 3.4 and Section 3.7). The free-AI estimate is not robust to adjustment for calendar time and is not reported as a finding (Section 3.6). Estimates for provider specialty describe provider cancellation behaviour rather than user behaviour (Section 3.4) and, because the extract contains no de-identified individual provider key, may substantially reflect clustering of cancellation practice within individual providers rather than any property of the specialty (Section 3.2 and Section 4.4).

## Data Availability

The de-identified dataset analysed in the present study is owned and controlled by the Talbinah platform (the data controller); the corresponding author accessed it as an external researcher under a data-governance agreement and does not have independent authority to release it. Restrictions therefore apply: the raw dataset is not publicly available and any release requires Talbinah’s authorisation. Researchers may direct access requests to the corresponding author, who will forward them to the platform’s data-governance team; requests are considered on a case-by-case basis subject to a data-governance agreement. To support independent scrutiny of everything that does not require the raw records, the complete exclusion cascade is reported stage by stage, with the number of bookings and unique users remaining at each stage, in Section 2.3 and in the flow diagram in Figure 1; the derivation rules for every covariate, including the prior-attendance-history and calendar-time variables, the age and de-duplication cleaning rules, and the exact reference category and coding of every model term, are fully specified in Section 2.3, Section 2.4, Section 2.5 and Section 2.6; and a completed STROBE checklist is provided as Supplementary Material. The de-identified analysis code and the full model specification on the log-odds scale, including coefficients and standard errors for the models summarised in Section 3.3, Section 3.4, Section 3.5, Section 3.6, Section 3.7 and Section 3.8, are available from the corresponding author on reasonable request.

## Supplementary Materials

The following supporting information can be downloaded at: https://www.mdpi.com/article/10.3390/healthcare14172672/s1, A completed STROBE cohort-study checklist mapping every recommended reporting item to the specific section of this manuscript in which it is addressed.
