# Supplementary material for: Factors Associated with Appointment Cancellation on a Commercial Digital Mental Health Platform: A Retrospective Cohort Study in Saudi Arabia
Source: Healthcare (Basel). 2026 Aug 22;14(17):2672. doi: 10.3390/healthcare14172672 (PMC13565555; doi:10.3390/healthcare14172672)
Supplement: Supplementary file 1 [file healthcare-14-02672-s001.zip › healthcare-4482172-supplementary.pdf]

STROBE Statement — Checklist of items to include in reports of cohort studies

Manuscript healthcare-4482172

Factors Associated with Appointment Cancellation on a Commercial Digital Mental Health Platform: A Retrospective Cohort Study in Saudi Arabia

Abdulmajeed A. Alkhamees

This checklist is based on the STROBE Statement for cohort studies (von Elm et al., 2007; see manuscript reference list). Item 6b, 12d, 14c and 16c are marked “Not applicable” with a brief, study-specific explanation, as recommended by the STROBE checklist for studies where these items do not arise. All section numbers refer to the accompanying revised manuscript.

Section/Topic	Item	Recommendation	Reported on page/section
Title and abstract	1a	Indicate the study's design with a commonly used term in the title or the abstract.	Title (“A Retrospective Cohort Study”); Abstract
	1b	Provide in the abstract an informative and balanced summary of what was done and what was found.	Abstract
Introduction — Background/rationale	2	Explain the scientific background and rationale for the investigation being reported.	Section 1 (Introduction)
Introduction — Objectives	3	State specific objectives, including any prespecified hypotheses.	Section 1, closing paragraph
Methods — Study design	4	Present key elements of study design early in the paper.	Section 2.2 (Study Design and Data Source)
Methods — Setting	5	Describe the setting, locations, and relevant dates, including periods of recruitment, exposure, follow-up, and data collection.	Sections 2.1–2.2 (platform description; October 2023–April 2026 window)
Methods — Participants	6a	Give the eligibility criteria, and the sources and methods of selection of participants.	Section 2.3 (Inclusion and Exclusion Criteria); Figure 1 (flow diagram)
	6b	For matched studies, give matching criteria and number of exposed and unexposed.	Not applicable — this is a secondary analysis of routinely collected booking records, not a matched exposed/unexposed cohort design.
Methods — Variables	7	Clearly define all outcomes, exposures, predictors, potential confounders, and effect modifiers. Give diagnostic criteria, if applicable.	Sections 2.4 (Outcome Definition) and 2.5 (Study Variables)
Methods — Data sources/measurement	8	For each variable of interest, give sources of data and details of methods of assessment (measurement). Describe comparability of assessment methods if more than one group.	Sections 2.2 and 2.5; Section 2.6 (derivation of prior-history and calendar covariates)
Methods — Bias	9	Describe any efforts to address potential sources of bias.	Sections 2.3 (age-plausibility restriction), 2.6 (cluster-robust standard errors; Firth penalisation for sparse cells), 3.4–3.8 (cause-specific decomposition, splines, calendar adjustment, and overlap diagnostics undertaken specifically to address potential confounding and outcome heterogeneity), and Section 4.4 (Limitations)
Methods — Study size	10	Explain how the study size was arrived at.	Section 2.3 (the analytic sample is

Section/Topic	Item	Recommendation	Reported on page/section
			the full population of eligible bookings in the extract; no a priori sample-size calculation was performed, as noted in Section 2.6)
Methods — Quantitative variables	11	Explain how quantitative variables were handled in the analyses. If applicable, describe which groupings were chosen and why.	Section 2.5 (age and lead-time categorisation for the primary model) and Section 3.3 (restricted-cubic-spline treatment of age and lead time as continuous variables, and the rationale for testing linearity)
Methods — Statistical methods (a)	12a	Describe all statistical methods, including those used to control for confounding.	Section 2.6 (Statistical Analysis)
Methods — Statistical methods (b)	12b	Describe any methods used to examine subgroups and interactions.	Section 3.4 (cause-specific models by recorded cancellation initiator) and Section 3.8 (structured-subgroup sensitivity analyses excluding Free-AI and emergency session types)
Methods — Statistical methods (c)	12c	Explain how missing data were addressed.	Section 2.3 (exclusion of records with missing age or non-binary recorded gender) and Section 2.5 (retention of “Unknown” device type as an explicit category, with the corresponding wording in Section 2.5 not inferring a missing-data mechanism)
Methods — Statistical methods (d)	12d	Cohort study—If applicable, explain how loss to follow-up was addressed.	Not applicable — the analysis is a cross-sectional-style analysis of a closed, retrospectively extracted set of booking records with a binary outcome (cancelled vs. completed) recorded at the time of extraction; there is no loss to follow-up in the prospective sense.
Methods — Statistical methods (e)	12e	Describe any sensitivity analyses.	Section 3.7 (alternative outcome definitions: the restrictive was_cancelled flag and a strictly concordant definition) and Section 3.8 (exclusion of structurally dependent session categories)
Results — Participants (a)	13a	Report the numbers of individuals at each stage of the study—e.g. numbers potentially eligible, examined for eligibility, confirmed eligible, included in the study, completing follow-up, and analysed.	Figure 1 (STROBE-style flow diagram, all six exclusion steps with booking and user counts at each stage) and Section 2.3
Results — Participants (b)	13b	Give reasons for non-participation at each stage.	Section 2.3 and Figure 1 (each exclusion step is individually disaggregated and quantified, including the duplicate-row-emission correction described in Section 2.3)

Section/Topic	Item	Recommendation	Reported on page/section
Results — Participants (c)	13c	Consider use of a flow diagram.	Figure 1
Results — Descriptive data (a)	14a	Give characteristics of study participants (e.g. demographic, clinical, social) and information on exposures and potential confounders.	Table 1 and Section 3.1
Results — Descriptive data (b)	14b	Indicate the number of participants with missing data for each variable of interest.	Section 2.3 (missing-data counts by exclusion category) and the device-type missingness figure reported in Section 2.3
Results — Descriptive data (c)	14c	Cohort study—Summarise follow-up time (e.g., average and total amount).	Not applicable — see 12d above; there is no follow-up interval to summarise.
Results — Outcome data	15	Cohort study—Report numbers of outcome events or summary measures over time.	Table 1 and Section 3.1 (crude cancellation counts and rate); Section 3.4 (outcome events by recorded cancellation initiator)
Results — Main results (a)	16a	Give unadjusted estimates and, if applicable, confounder-adjusted estimates and their precision. Make clear which confounders were adjusted for and why they were included.	Table 2 (adjusted odds ratios with 95% confidence intervals and p-values) and Section 3.2; the covariate set and rationale are given in Section 2.5
Results — Main results (b)	16b	Report category boundaries when continuous variables were categorized.	Section 2.5 (lead-time and age categories) and Section 3.3 (spline-based continuous treatment and the linearity tests that motivated retaining categorical lead time and age in the primary model)
Results — Main results (c)	16c	If relevant, consider translating estimates of relative risk into absolute risk for a meaningful time period.	Not applicable — the outcome is binary cancellation without a defined follow-up interval; absolute within-category cancellation rates with 95% Wilson confidence intervals are reported in Table 1 in place of a time-based absolute-risk translation.
Results — Other analyses	17	Report other analyses done—e.g. analyses of subgroups and interactions, and sensitivity analyses.	Sections 3.3–3.9 (restricted cubic splines; cause-specific decomposition; prior-attendance history; calendar-time adjustment; alternative outcome definitions; covariate overlap and sparsity diagnostics; exploratory model-fit statistics)
Discussion — Key results	18	Summarise key results with reference to study objectives.	Section 4.1 (Principal Findings)
Discussion — Limitations	19	Discuss limitations of the study, taking into account sources of potential bias or imprecision. Discuss both direction and magnitude of any potential bias.	Section 4.4 (Limitations)
Discussion — Interpretation	20	Give a cautious overall interpretation considering objectives, limitations, multiplicity of analyses, results from similar studies, and other relevant evidence.	Sections 4.1–4.3

**Section/Topic**	**Ite m**	**Recommendation**	**Reported on page/section**
Discussion — Generalisability	21	Discuss the generalisability (external validity) of the study results.	Section 4.4 (Limitations) and Section 4.3 (Implications for Platform Operations)
Other information — Funding	22	Give the source of funding and the role of the funders for the present study and, if applicable, for the original study on which the present article is based.	Funding statement (back matter)

Note: An explanation and elaboration article discusses each checklist item and gives methodological background and published examples of transparent reporting. The STROBE checklist is best used in conjunction with this article (freely available on the Web sites of PLoS Medicine at http://www.plosmedicine.org/, Annals of Internal Medicine at http://www.annals.org/, and Epidemiology at http://www.epidem.com/). Information on the STROBE Initiative is available at http://www.strobe-statement.org.
